# Research Progress and Application Prospects of Plant Fibers in Geopolymer Concrete: A Review

**DOI:** 10.3390/ma18102342

**Published:** 2025-05-17

**Authors:** Zijian Li, Jinjie Li, Weihua Lu, Yongxing Zhang

**Affiliations:** 1 School of Civil Engineering, Nanjing Forestry University, Nanjing 210037, China; 2 Jiangsu Provincial Highway Intelligent Detection and Low-Carbon Maintenance Engineering Research Center, Nanjing Forestry University, Nanjing 210037, China

**Keywords:** geopolymers, plant fibers, mechanical properties, durability

## Abstract

Plant fibers, characterized by their low density, renewable nature, and environmentally friendly characteristics, offer considerable potential as reinforcement materials in geopolymer composites. This review provides a critical and thorough examination of recent developments and emerging trends in plant fiber-reinforced geopolymer concrete (PFRGC). The paper commences by detailing the inherent characteristics of plant fibers and the mechanisms governing their interfacial adhesion with the geopolymer matrix, with specific emphasis on the impact of fiber surface modification on interface properties. The review offers a comprehensive investigation of the mechanical properties of plant fiber-reinforced geopolymer concrete, encompassing compressive strength, tensile strength, and toughness. Additionally, the paper examines the influence of plant fiber integration on the durability of geopolymer concrete, discussing improvements in freeze-thaw resistance, permeability, and carbonation resistance. In conclusion, this review highlights the prevailing challenges in the domain and provides insights into future developments of plant fiber-reinforced geopolymer concrete. An analysis was performed utilizing papers from 2000 to 2025 indexed in prominent databases including Web of Science, Scopus, and ScienceDirect to enhance the review. Integrating plant fibers into developing technologies, such as 3D printing of geo-polymer matrices, signifies a promising avenue for structural applications. It advocates that future research efforts should focus on enhancing fiber modification techniques, exploring novel fiber materials, and doing thorough assessments of long-term performance.

## 1. Introduction

Under the dual challenges of global climate change and resource shortening, the construction industry, as one of the major areas of resource consumption and carbon emission, is facing unprecedented pressure for transformation. The high energy consumption and large amount of CO_2_ emissions in the traditional cement production process have become an important obstacle to sustainable development [1,2]. Against this background, Geopolymer Concrete (GPC), as a new type of low-carbon and environmentally friendly cementitious material system, has received widespread attention due to its significant environmental benefits and excellent engineering performance.

GPC is mainly formed by the hardening of industrial wastes (e.g., fly ash, slag, etc.) rich in activated silica-aluminum through alkali-activated polymerization reaction [3]. Compared with traditional cement concrete, GPC not only exhibits excellent early strength development, better heat resistance and chemical stability, but also effectively solves the problem of industrial solid waste disposal and realizes the recycling of resources [3,4]. However, similar to traditional cement concrete, GPC has inherent defects such as high brittleness, low tensile strength, and poor impact resistance, which severely limit its application in structures that require high toughness and ductility [4]. Consequently, enhancing the mechanical properties of GPC, particularly its toughness and ductility, has emerged as a critical scientific challenge for facilitating its broader implementation.

Fiber reinforcement technology, as a mature and effective method of concrete modification, can significantly improve the crack resistance, toughness and ductility of composite materials by incorporating fiber materials with high tensile strength into the brittle matrix [5]. Traditional research on fiber-reinforced concrete mainly focuses on steel fibers and synthetic fibers (e.g., polypropylene fibers, carbon fibers, etc.). However, the production process of these synthetic fibers is usually accompanied by high energy consumption and environmental pollution, which contradicts the environmental protection concept of GPC [6]. The concept of sustainable development has become a hotspot for research on plant fibers. With the deepening of the concept of sustainable development, natural, renewable and environmentally friendly plant fibers have gradually become a research hot spot [7].

Plant fibers, including bamboo, hemp, coconut, sisal, and jute, possess diverse sources, affordability, low density, and high specific strength. Notably, their manufacturing and processing methods are characterized by low energy consumption and environmental sustainability [8]. The application of plant fibers to the reinforcement modification of GPC not only enhances the mechanical properties of GPC, but also further enhances its environmental sustainability and realizes the synergistic use of industrial waste and natural renewable resources, which is of great environmental and economic significance.

However, Plant Fiber Reinforced Geopolymer Concrete (PFRGC), as an emerging composite material, still faces many challenges in its performance regulation mechanism and engineering applications. Firstly, the hydrophilicity and porous structure of plant fibers may affect the workability and compactness of concrete; secondly, the stability and durability of plant fibers in high alkaline environments are of concern; furthermore, the interfacial bonding mechanism between the fibers and the geopolymer matrix and its effect on the performance of the composites have not been fully elucidated. The solution of these scientific issues is crucial for the performance optimization and engineering applications of PFRGC.

This review provides a thorough examination of the evolution of PFRGC, aimed at facilitating continued research and practical implementation by scientists, engineers, and practitioners in the discipline. The reviewed literature covers the period from 2000 to 2024 and was obtained from prominent databases including Web of Science, Scopus, and ScienceDirect, utilizing keyword combinations such as “plant fiber”, “natural fiber”, “geopolymer”, “geopolymer concrete”, “mechanical properties”, and “durability”. The review examines the interfacial interactions between plant fibers and the geopolymer matrix, methods for enhancing mechanical performance, and aspects linked to durability. It additionally examines the scientific and technical problems that persist and delineates possible directions for future research. This work aims to elucidate existing knowledge and enhance the design and implementation of PFRGC in engineering contexts by referencing a wide array of publications.

## 2. Interfacial Bonding Between Plant Fibers and Geopolymer Matrix

The interfacial adhesion between the plant fibers and the geopolymer matrix is essential for the composite’s overall performance. If the interfacial bonding is poor, the fibers will be easily debonded from the matrix when subjected to force, thus weakening the reinforcing effect of the composites. Thus, it is crucial to comprehensively examine the interfacial bonding performance between plant fibers and the geopolymer matrix, along with its assessment methodology.

The interfacial bonding property is an essential feature of fiber-reinforced composites, directly influencing the effectiveness of reinforcement. In plant fiber-reinforced geopolymer concrete, the qualities of interfacial bonding are especially critical. Compared to inorganic and synthetic fibers, plant fibers have unique properties that complicate their interfacial bonding behavior with the geopolymer matrix.

### 2.1. Types and Properties of Plant Fibers

Natural plant fiber is a kind of natural composite material with precise hierarchical structure, which can be divided into eight categories according to its source parts and usage characteristics: bast fiber (from the epidermis and phloem of plant stems), leaf fiber (extracted from leaf tissues), herbaceous fiber (obtained from graminaceous plants), seed fiber (obtained from the epidermis of the seed or endocarp), and root fiber (obtained from the root tissues of the plant), Fruit fibers (from the epidermis or endocarp of the fruit), stem core fibers (from the medullary part of the plant stem), and wood pulp fibers (separated from wood by chemical or mechanical processes).

The chemical composition primarily comprises cellulose (35–85%), hemicellulose (10–25%), lignin (5–30%), and trace quantities of organic constituents such as pectin and wax. These components construct a multilevel structural system from molecular to macroscopic scales by precise spatial organization, establishing a distinctive mechanical property transmission process.

From the structural level, the cell walls of plant fibers show a highly organized laminar structure [9]: the outermost layer is the primary wall, the inner one is the secondary wall consisting of S1, S2, and S3 layers, and the center is the lumen. The S2 layer, one of the three layers of the secondary cell wall, is crucial in determining the mechanical properties of natural fibers because of its significant thickness and highly ordered structure. It primarily comprises helically arranged cellulose microfibrils embedded in an amorphous matrix of lignin and hemicellulose, creating a natural fiber-reinforced composite. Cellulose, the principal structural component, consists of linear chains of β-1,4-linked D-glucose units, which constitute the repeating unit of the polymer with the chemical formula (C_6_H_10_O_5_)_n_. These chains consolidate into crystalline microfibrils, whose alignment within the S2 layer determines the tensile strength and rigidity of the fiber.

In this sophisticated structural system, hemicellulose is distributed among cellulose microfibrils in a disordered amorphous state, which provides secondary mechanical support while its hydrophilic character significantly influences the interfacial interactions between the fibers and the geopolymer matrix. Lignin, on the other hand, acts as a natural binder to cement the entire structure into a unified whole, and its complex network of aromatic polymers not only enhances the rigidity and structural stability of the fibers, but also affects the durability of the composites by regulating the water absorption. The bast fibers show a regular longitudinal texture on the surface, the leaf fibers have a helical vascular bundle structure, and the stalk fibers exhibit a porous network structure. This structural diversity stems from the specific assembly of cell wall components during biosynthesis, which directly affects the reinforcement of fibers in composites.

The stability of plant fiber characteristics is substantially affected by environmental variables. As can be seen from the figure, the strength enhancement, water absorption, thermal degradation and biodegradation properties of fibers are all closely related to their component structures and show obvious hierarchical nature: lignin, hemicellulose and cellulose are involved in these processes sequentially, forming a complex performance response mechanism. The multilayer structure-property relationship serves as a crucial framework for comprehending and enhancing the utilization of plant fibers in geopolymer reinforcement systems.

Considering the fundamental structural attributes and chemical composition of plant fibers, those derived from various organ origins exhibit distinct structural characteristics and performance due to variations in their growth environments and physiological functions. Therefore, a comprehensive analysis of the differences in characteristics among common fiber types is essential from an organ classification perspective. According to the origin of plant organs, the common fiber types and their characteristics are as shown in [Table 1].

Among the many plant fibers, the bast fiber class shows the most excellent mechanical properties. Flax fiber, for example, has tensile strength up to 1800 MPa, elasticity modulus up to 103 GPa [10,11], which is closely related to its highly oriented cellulose molecular structure. In situ tensile test results show that flax fiber in the force process shows obvious graded damage characteristics; this progressive destruction mechanism is conducive to improving the toughness of the composite material. Leaf fiber class has a high elongation at break due to its unique helical structure, and exhibits excellent energy absorption capacity under dynamic loading.

**Figure 1:**
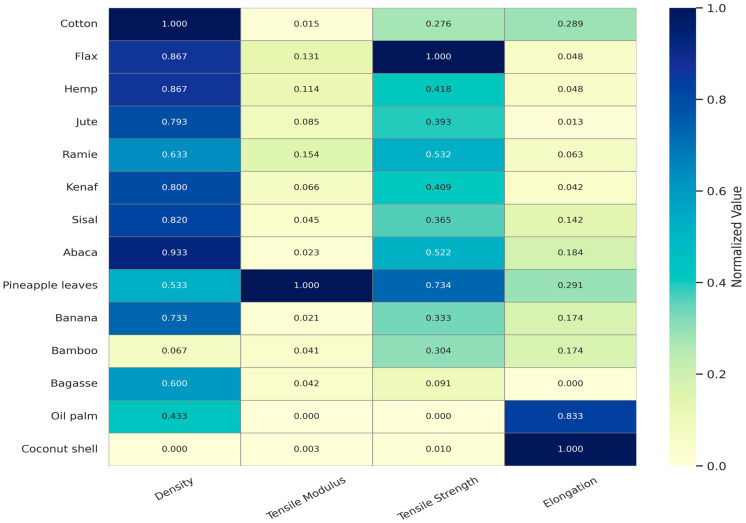
Normalized comparison of key mechanical properties among various plant fibers.

The subsequent heatmap displays a normalized comparison of four essential mechanical properties—density, tensile modulus, tensile strength, and elongation at break—across fourteen prevalent plant fibers [Figure 1]. Each statistic was normalized to a range of 0 to 1 to remove unit discrepancies and emphasize relative performance. The deeper hues in the spectrum signify elevated values. This visualization technique has been effective in enabling an intuitive assessment of fiber appropriateness for reinforcing in geopolymer.

**Figure 2:**
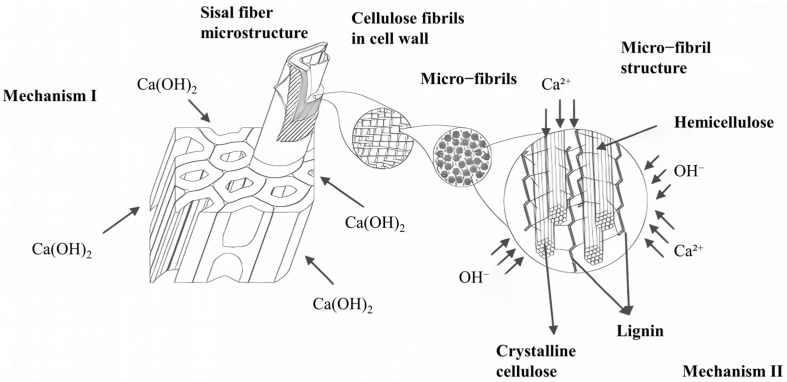
Degradation mechanism of plant fibers proposed [13].

However, the intrinsic properties of plant fibers pose a serious challenge to interfacial stability. The primary problem is the dimensional instability due to the hydrophilic nature of the fibers, a volume change that causes interfacial stress concentration and microscopic crack formation. More critically, Melo Filho et al. [13] revealed two main degradation mechanisms of plant fibers in alkaline environments [Figure 2]: one is the process of mineralization by deposition of calcium hydroxide crystals on the surface of the fibers, which significantly reduces the active cellulose content of the fibers, weakening their reinforcing effect; and the other is the process of hemicellulose and chemical degradation of lignin in an alkaline environment, and this component dissociation process destroys the integrity of the fiber, which in turn affects the long-term stability of interfacial bonding. The molecular chains of cellulose consist of crystalline and amorphous areas, which exhibit reduced stability in alkaline conditions [14].

The morphological attributes of the fibers (e.g., aspect ratio, cross-sectional geometry, surface texture) substantially influence their properties. The degree of processing of plant fibers directly affects their reinforcing capacity in geopolymers. Korniejenko et al. [15] conducted a comprehensive study on the effects of various fiber types and processing stages on the characteristics of fly ash-based geopolymers. The inclusion of unrefined fibers, marked by a rough texture, presents a difficulty in the mixing process owing to their propensity for uneven dispersion. This may result in the development of stress concentration zones, hence diminishing the composite’s overall performance. In contrast, finely treated fibers demonstrate improved distribution and establish stronger interfacial interactions with the matrix, resulting in significant enhancements in flexural and compressive properties.

In conclusion, all these factors affect the long-term stability of interfacial bonding, highlighting the need for a systematic modification strategy. Therefore, a systematic modification strategy is required to optimize the fiber-matrix interfacial properties, which will be discussed in detail in the subsequent sections.

### 2.2. Fiber Surface Treatment

Fiber surface treatment is a prevalent technique employed to enhance adhesion at the fiber-matrix contact. Physically or chemically altering the surface of plant fibers can enhance their surface qualities, hence improving binding strength with the geopolymer matrix.

#### 2.2.1. Alkali Treatment

Alkali treatment is a straightforward and efficient technique for fiber surface modification. Soaking plant fibers in alkaline solutions, such as sodium hydroxide and calcium hydroxide, effectively eliminates waxes, oils, and other surface contaminants, enhances the roughness of the fiber surface, and improves mechanical interlocking. Simultaneously, alkali treatment can partially eliminate lignin and hemicellulose from the cellulose surface, thereby exposing additional cellulose and augmenting the quantity of hydroxyl groups on the fiber surface. This enhancement facilitates chemical reactions with the geopolymer matrix and improves the mechanical properties and durability of the composites [16,17].

Alkali treatment process parameters such as lye concentration, treatment temperature, and time have a significant effect on the modification effect [18,19]. Bast fibers, including jute and flax, are often treated with a 5–8% NaOH solution for 1–4 h at ambient temperature, whereas woody fibers, such as bamboo and rice straw, generally require treatment at 60–80 °C. Excessive alkali treatment can cause hydrolytic destruction of cellulose molecular chains, leading to reduced fiber strength; thus, optimizing the treatment conditions is essential [20].

The enhancement of interfacial characteristics between plant fibers and geopolymer through alkali treatment was evident. Single-fiber pull-out test findings indicated that treatment with 5% NaOH enhanced the interfacial shear strength between fibers and the GPC matrix by a factor of 2.5 [21]. The 5% NaOH-treated fiber-reinforced composites shown notable improvements in overall performance, with tensile and flexural strengths rising by 8.14% and 7.88%, respectively [22]. Under elevated temperature conditions (100 °C, 1% NaOH, 1 h), the treated date palm fiber composites demonstrated superior mechanical properties, including enhanced impact strength, tensile strength, tensile modulus, and flexural strength, along with improved fiber dispersion within the matrix [23]. In addition, alkali treatment is effective in reducing the degradation rate of plant fibers in alkaline geopolymer environments, improving the durability and dimensional stability of the composites.

Nonetheless, a contradiction exists regarding the alkali treatment technique: whereas the monofilament tensile strength of the treated fibers often diminishes, composites fabricated from these fibers exhibit enhanced mechanical properties [24]. The research performed by Fernandes et al. [25] offers significant insights into the comprehension of this phenomenon. Sisal fibers, despite a decrease in strength from the partial extraction of hemicellulose and lignin, achieved excellent mechanical qualities when included into composites at a concentration of 10 wt%. The essence of this apparent contradiction is in the augmented surface roughness resulting from the alkali treatment, which markedly improves the mechanical interlocking at the fiber-matrix interface [26]. Optimization of surface morphology and improvement of interfacial structure may contribute more to the composite properties than the single fiber strength.

In summary, the alkali treatment method significantly enhances the interfacial compatibility and bonding characteristics between plant fibers and the geopolymer matrix via various physicochemical mechanisms. Nevertheless, the impact of a singular alkali treatment on certain plant fibers may nevertheless exhibit constraints.

#### 2.2.2. Silane Coupling Agent Treatment

Silane coupling agent modification, as a key technology for surface treatment of plant fibers, significantly improves fiber-substrate interfacial properties through molecular bridging mechanisms. The molecular structure of silane coupling agents is usually expressed as R-Si(OR’)_3_, where R represents an organic functional group capable of reacting with plant fibers, and the Si(OR’)_3_ portion is hydrolyzed to form a chemical bond with the geopolymer matrix [27,28].

The molecular mechanism of silane modification includes two key steps, hydrolysis and condensation: silane molecules are first hydrolyzed in aqueous solution to generate Si-OH groups, which subsequently condense with the hydroxyl groups on the fiber surface to form Si-O-C bonds, and at the same time, self-condensation between adjacent Si-OH groups to form a Si-O-Si network structure [28,29,30]. This silicone crosslinked network not only improves the hydrophobicity of the fibers, but also establishes a stable chemical connection between the organic fibers and the inorganic matrix.

The application effects of different types of silane coupling agents are different. KH570 significantly enhanced the interfacial integrity of the system by dehydration condensation reaction with free water or adsorbed water in the matrix [31]. Wu and Zuo’s research [32] showed that in the plant cellulose/waste tire powder composite system, Si69 showed a better interfacial enhancement effect than KH550 at the optimal addition amount. The 5% KH550 treatment can increase the energy absorption capacity of the fiber reinforced system by 13.8% and improve the cracking behavior [33]. According to Zhao et al. [34], KH550 showed the most significant enhancement in both sisal fiber and matrix properties due to its amino functional group, which contributes to superior hydrophobicity, degradation resistance, barrier performance, and interfacial bonding. KH792 ranked second in efficacy, while KH560 and KH570 showed relatively weaker and inconsistent effects. Liu et al. [35] found that KH560, when used as an intermediate agent in nano-SiO_2_ surface modification, facilitated strong adhesion between nanoparticles and fiber surfaces by inducing abundant hydrogen bonds at the interface. Furthermore, nano-SiO_2_ could interact with the N-A-S-H gel matrix to form ionic and covalent Si–O–Si bonds, thereby significantly enhancing the interfacial bonding strength.

Silane treatment has a different mechanism of action compared to alkali treatment: alkali treatment improves interfacial bonding mainly by increasing surface roughness and active sites, whereas silane treatment realizes chemical bonding at the molecular scale. The alkali-silane composite modification process has been shown to fully utilize the synergistic effect of the two methods, and Orue and Jauregi et al. [21] showed that pretreatment with a low concentration (2–5%) of NaOH to remove surface waxes and activate hydroxyls, followed by silane treatment, increased the bond strength of the fiber-substrate interface by a factor of 2–3. Similarly, Asim et al. [30] confirmed that a 2% silane solution treatment for 3 h significantly improved the tensile strength and interfacial adhesion of pineapple and jute fibers. SEM, FTIR, and tensile tests confirmed the removal of non-cellulosic components and enhanced compatibility between the fibers and the geopolymer matrix.

#### 2.2.3. Polymer Coating

Polymer cladding, a novel fiber surface modification technology, provides an innovative solution to the durability of fibers in geopolymer environments by constructing a continuous or semi-continuous polymer protective layer on the surface of plant fibers. This surface engineering strategy has been shown to significantly reduce water absorption while forming a flexible transition zone between fibers and the matrix, thereby optimizing interfacial stress distribution. Common cladding materials include epoxy resins, polyurethanes, acrylic resins, and other polymers, the selection of which is primarily based on compatibility with fibers and the matrix, as well as target performance requirements.

Research has demonstrated that the application of fiber surface protection, encompassing methods such as polymer coating and alkali treatment, constitutes a highly efficacious approach to mitigating the degradation of plant fibers within alkaline contexts [36]. The fundamental basis of this protective effect is the construction of a barrier layer that effectively mitigates the erosion of fibers by alkaline media.

Li et al. [37] elucidate the mechanism of action underlying polymer encapsulating technology. The epoxy glue penetrates the fiber lumen to create a reinforcing structure and effectively inhibits crack propagation. The occupation of the lumen space by the hydrophobic epoxy resin markedly enhances the water resistance of the fiber. The experimental findings indicate that this treatment can significantly enhance the mechanical properties of the composites, including tensile strength, bending strength, and impact strength, while efficiently regulating the water absorption rate.

However, the relatively high cost of raw materials and the more complex process have limited its large-scale application to some extent.

#### 2.2.4. Other Surface Treatment Methods

In addition to the aforementioned methods, there are other fiber surface treatment methods, including but not limited to plasma treatment, graft copolymerization, and microwave treatment [38,39]. The combination of multiple treatments often exhibits a synergistic effect, resulting in a modification that is often superior to that of a single treatment. Each of these methods possesses its characteristics, with a different scope of application and effects. Therefore, the selection of an appropriate method must be made according to the specific fiber type and application requirements.

It is important to acknowledge the existence of discrepancies in the mechanisms through which diverse surface treatments influence the performance of fiber-GPC composites. Their applicability is also closely related to specific fiber types, matrix ratios, and application environments. Alkali treatment remains the most widely used method due to its simplicity, efficiency, and significant effect. Polymer coating and silane coupling agent treatment are dominant in high-performance applications. Emerging technologies, such as plasma treatment and microwave treatment, show promise for development in specific scenarios.

### 2.3. Geopolymer Matrix Modification Methods

The design of matrix modification is crucial and serves as another important way to improve the performance of fiber-reinforced composites. Optimizing the mixing ratio can enhance the compatibility of the fiber-matrix interface, encompassing precursor composition, alkali-activated ratio, and liquid-solid ratio, among other variables [40]. The Si/Al molar ratio exerts a substantial influence on the structural characteristics of geopolymer networks and the properties that define their interfacial interactions [41].

The durability of fibers can be improved via matrix modification, with volcanic ash materials providing a unique benefit as a cement alternative. The consumption of calcium hydroxide by the matrix pore solution results in the formation of C-S-H/C-A-S-H, leading to a reduction in pH, which in turn mitigates the degradation of plant fibers [42]. Wei and Meyer’s research [43] demonstrated that the incorporation of 30% rice husk ash, instead of Portland cement, resulted in a decline of the substrate pH from 13.75 to 12.7. This adjustment effectively mitigated the degradation of sisal fibers. With regard to the optimization of raw material combinations, Gholampour et al. [44]. The study’s findings revealed that the ratio of fly ash (FA) to blast furnace slag (GGBS) significantly influenced the strength development and microstructure of GPC. In both the F80G20 and F50G50 ratios, increased GGBS content was shown to facilitate C-S-H gel formation, thus improving concrete densities and strengthening fiber-matrix interface bonding. This conclusion is corroborated by the research conducted by Poletanovic et al. [45]. This finding further corroborates the study’s conclusions. Through a comparative analysis of alkali-activated mortars comprising solely fly ash (FA1) and fly ash-slag mixtures (FA2S), it was ascertained that the gels generated by these disparate precursors exerted a substantial influence on the mechanical properties and pore structure of the materials. The incorporation of hemp fibers led to a substantial enhancement in the energy absorption capacity of the alkali-activated materials, a phenomenon that was particularly evident in brittle matrices. Furthermore, the study indicated that the incorporation of fibers exerted a negligible effect on density and water absorption. Nonetheless, it enhanced the compressive strength of the fly ash-based mortar, an effect principally ascribed to the improved interfacial bonding and frictional stress transmission mechanisms.

The optimization of the alkali-activated system is a pivotal factor influencing the performance of plant fiber-reinforced geopolymers. Phoo-ngernkham et al. [46] The Na_2_SiO_3_/NaOH composite activator was found to significantly improve the densification of the interfacial zone in comparison with single NaOH or KOH systems. This enhancement in performance is attributed chiefly to the additional Si source provided by the soluble silicate, which promotes the formation of a Si-O-Si network structure, optimizes the pore structure characteristics of the matrix, and enhances the mechanical occlusion with the fiber surface.

The type and concentration of alkali activators have been demonstrated to exert a substantial influence on material properties. It has been demonstrated that higher concentrations of sodium hydroxide, while increasing the compressive strength, concomitantly reduce the workability of the material [47]. Regarding activator selection, Sá Ribeiro et al. [48] reported that sodium-based systems exhibit superior performance compared to potassium-based counterparts. In their study, a sodium-metakaolin geopolymer incorporating 20 wt% fine sand and 5 wt% bamboo fibers achieved a 32% higher flexural strength than its potassium-based equivalent. Toledo Filho et al. [12] determined through immersion experiments that the tensile strength of fibers was entirely diminished after 300 days in a calcium hydroxide solution, whereas the strength of sisal and coir fibers decreased by 72.7% and 60.9%, respectively, in a sodium hydroxide solution. This finding indicates that the pore solution of traditional cementitious materials exerts a more pronounced erosive effect on plant fibers compared to the NaOH-activated alkali-activated system. M. Jegan et al. [49] The study demonstrated that the judicious selection of alkali activators has the potential to enhance the interfacial interaction between the fibers and the geopolymer matrix. Moreover, it was demonstrated that this selection enables the fibers to fulfill both reinforcing and bonding roles in the concrete structure.

The liquid-solid ratio and the curing conditions have been demonstrated to exert a substantial influence on interfacial properties. Silva et al. [50] found that. In the range of observation, the liquid-solid ratio exhibited a decrease from 0.29 to 0.27. There was a significant increase in the compressive strength of the samples. However, Li et al. [51] A substantial body of research has demonstrated that elevated liquid-solid ratios result in a decline in compression and splitting tensile strength. This phenomenon is predominantly ascribed to the augmentation of material porosity and the diminution of interfacial compactness that accompany higher liquid-solid ratios. Furthermore, curing time and temperature have been shown to exert a substantial influence on material properties [50]. The application of heat during curing has been shown to enhance and expedite the dissolution of silica and alumina in precursor materials [52]. Consequently, the density of the material is increased, and the hardening process is accelerated.

Furthermore, nanomineral admixtures have been shown to enhance the microstructure and interfacial properties of the matrix through the microaggregate effect and volcanic ash reaction. Asaedi et al. [53] The research demonstrated that the toughness of the fly ash-based polymer matrix enhanced by roughly 58% as the concentration of silica nanoparticles rose from 1.0 wt% to 2.0 wt%. The improvement in performance is mainly due to the increased density (reduced porosity) resulting from the elevated gel content, which enhances the interfacial bonding between the fibers and the matrix. The synergistic application of multi-component mineral dopants, such as the integration of nano-SiO_2_ with nano-Al_2_O_3_, has been shown to enhance the Si/Al ratio and particle gradation. This combination has demonstrated a significant interfacial increase relative to a single component [54]. The selection and dosage optimization of dopants must include their compatibility with matrix components and their effect on workability.

### 2.4. Microstructural Characteristics of Interfacial Transition Zones (ITZ)

The Interfacial Transition Zone (ITZ) between plant fibers and the geopolymer matrix is a crucial area influencing the characteristics of composites. The microstructure and characteristics of the plant directly influence the composite’s overall performance. This interfacial contact exhibits distinct multi-scale characteristics: At the nanoscale, the plentiful hydroxyl functional groups on the fiber surface establish molecular interfacial connections with the silica-alumina-oxygen complexes in the matrix via covalent and hydrogen bonding. At the micrometer scale, the distinctive morphological characteristics on the fiber surface, such as grooves and pores, offer mechanical occlusion sites to the matrix, thereby improving interfacial anchoring. At the macroscopic scale, the spatial orientation and network structure of the fibers directly influence the efficiency and uniformity of stress transfer within the composites. The scale, spatial orientation distribution, and network structure of fibers directly influence the efficiency and homogeneity of stress transfer in composites.

ITZ formation in geopolymer systems is distinct from the process in conventional cementitious materials and is characterized by unique properties [55]. The surface of plant fibers exhibits partial solubility in hemicellulose and lignin. In an alkaline environment, hemicellulose and lignin on the surface of plant fibers are partially solubilized, creating a distinct “dissolution layer”. Simultaneously, the hydroxyl groups on the fiber surface engage in an ion exchange reaction with the silica-alumina-oxygen tetrahedra, facilitating the heterogeneous nucleation of alumino-silicate gels [56].

The interfacial enhancement mechanism is predominantly composed of chemical bonding and physico-mechanical occlusion [57]. The formation of chemical bonds is predominantly facilitated through the establishment of hydrogen, ionic, and covalent bonds (after surface modification) between hydroxyl groups located on the fiber surface and the geopolymer network [29]. In contrast, physical-mechanical occlusion is attributed to factors such as fiber surface roughness, mechanical locking of matrix products, and interfacial stress distribution. The formation of a robust interfacial bond between cellulose and the matrix is crucial for achieving optimal occlusion of the spiny or lamellar structure of the fiber surface with the matrix [58].

**Figure 3:**
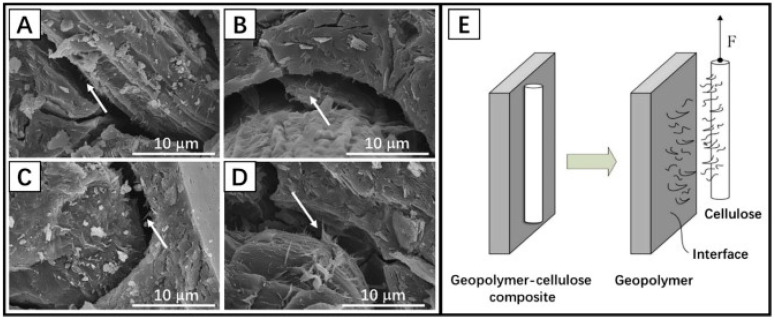
SEM images of the fiber–geopolymer interface (**A**–**D**). Arrows indicate embedded cellulose fiber fragments—mainly sheet- or needle-like—anchored in the matrix after flexural loading. (**E**) Schematic illustration of the bonding mechanism between cellulose fibers and the geopolymer matrix [16].

The factors that influence interfacial properties encompass environmental factors, such as temperature, humidity, and pH, as well as material factors, including fiber type and morphology, matrix composition, and maintenance conditions [59,60,61,62]. Wang et al. [61] has been determined that the proper conditioning temperature and relative humidity are conducive to the development of interfacial structure. Conversely, elevated pH levels (greater than 13) have been observed to potentially induce alkali corrosion of fibers and compromise the long-term stability of the interface [14]. The impact of fiber type and morphology on the formation of interfacial structures has been demonstrated to be significant. For instance, bast fibers, such as those found in flax and jute, have been observed to exhibit superior interfacial bonding properties due to their higher surface roughness [63]. Indeed, as Ye et al. [16], the structural characteristics of the interface between the geopolymer matrix and the all-cellulose fibers were revealed [Figure 3]. The spiny or lamellar structures on the cellulose surface were embedded into the interface of the geopolymer matrix, forming an effective mechanical anchorage point. Good interfacial bonding between cellulose and matrix was still formed in strong alkaline environments, and no obvious cellulose degradation was observed [58].

The microstructural modulation of the interfacial transition zone establishes a microscopic foundation for enhancing the overall performance of the composites. By systematically optimizing the structure of the interfacial zone, the mechanical and durability properties of the composites can be significantly enhanced. The effectiveness of these microstructure modulation strategies must be verified by scientific interfacial bonding performance evaluation methods.

### 2.5. Methods for Evaluating Interfacial Bonding Properties

The systematic evaluation of interfacial bonding properties is important for understanding the mechanical behavior and optimizing the interface design of fiber-reinforced geopolymer composites (FRGPC). Based on the multiscale characteristics of interfacial interaction, its evaluation system covers macroscopic mechanical properties, microstructural features and their interrelationships to form a complete interface characterization methodology.

**Figure 4:**
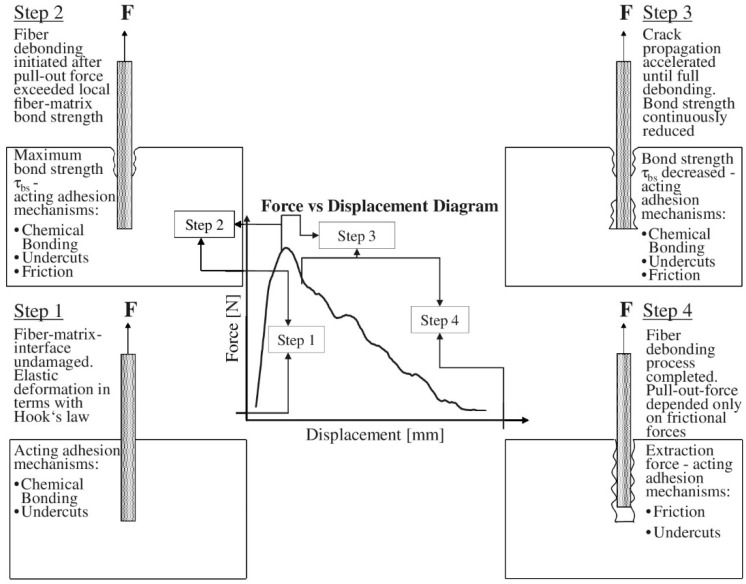
Schematic description of single fiber pull-out test (SFPT) steps [64].

The single fiber pull-out test (SFPT) is considered the most representative standard approach. The extensive utilization of it might be ascribed to its significant standardization and data dependability. The principal failure mechanisms for both species have been recognized as fiber fracture and matrix pullout. The failure mechanism of the fiber-matrix interface was methodically assessed, and the interfacial shear strength was measured by meticulously regulating the fiber embedment length (10–25 mm) and the pull-out rate (0.5–2 mm/min). The standard load-displacement curve is segmented into four phases: the linear elastic phase (Step 1, 0–0.2 mm), which indicates initial stiffness and elastic deformation as dictated by Hooke’s law; the debonding phase (Step 2, 0.2–0.3mm) The contact begins to deteriorate when the tensile force attains the maximal bonding strength (*τ_bs_*). This is succeeded by the fracture propagation phase (Step 3, 0.3–0.5 mm), during which interfacial cracks expedite their growth, bonding strength diminishes, and friction is added. Ultimately, the friction stage (Step 4, >0.5 mm) is attained, wherein the fiber is entirely extracted, and the remaining load-bearing capacity depends exclusively on friction [Figure 4]. The determination of interfacial shear strength is conducted via the examination of curve attributes, as specified in the following formula:

(1) $$\tau =\frac{F}{\pi dL}$$

In this formula, *F* denotes the maximum pull-out force, which is to be determined through predictive tests. *d* represents the fiber diameter, and *L* signifies the effective burial length.

Single-fiber diagonal pull tests were conducted by varying the fiber inclination angle (0–45°) to evaluate the mechanical response of the interface under composite stresses. The most significant increase in interface strength was found at an inclination angle of 15–30°, which is mainly attributed to the contribution of the fiber “wedge effect”.

The fiber critical length method is predicated on the Kelly-Tyson model.

(2) $$\tau =\frac{\sigma f\cdot d}{2Lc}$$

In this equation, *σ_f_* is the fiber tensile strength, which is to be determined by predictive tests, *d* is the fiber diameter, and *L_c_* is the critical length.

The method involves the use of fibers of varying lengths, which are mixed into the matrix. The statistical distribution of fiber lengths post-fracture is subsequently determined to ascertain the critical length. Fiber breakage occurs when the fiber length exceeds the critical length, and interfacial debonding occurs when it is less than the critical length. A significant advantage of this method is that it enables the simultaneous testing of a large number of fibers, thereby facilitating the acquisition of statistically significant interfacial property data. This method is particularly well-suited for short-fiber-reinforced systems.

Conversely, the three-point bending test appraises the interfacial action from the vantage point of overall performance. The measurement of load-deflection curves enables the determination of critical parameters, including initial cracking strength (*σ_fc_*), ultimate strength (*σ_u_*), and toughness index (*TI*). When these parameters are considered in conjunction with the crack extension mode and fiber bridging behavior, a comprehensive evaluation of the effect of interfacial modification can be performed.

**Figure 5:**
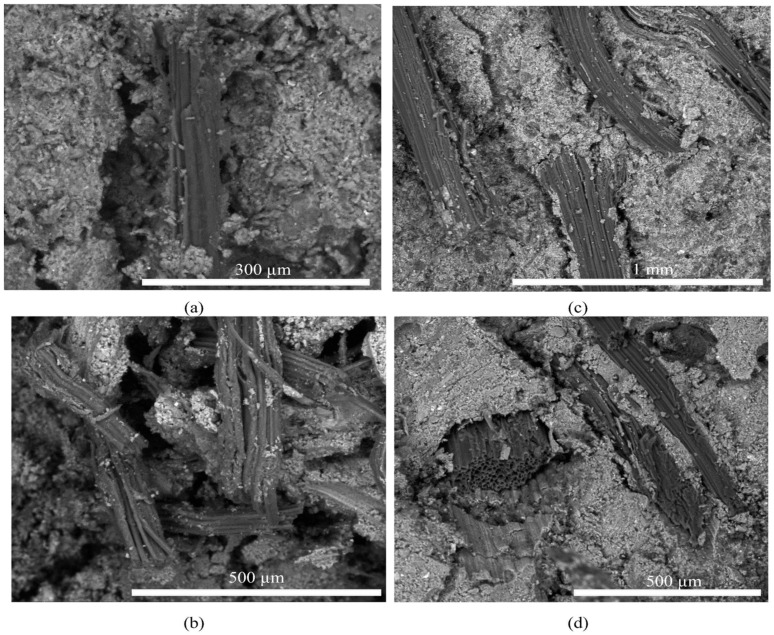
SEM micrographs of broken FRGCs: (**a**) Fiber-matrix bonding in jute-FRGC (2% wt%); (**b**) Failure mechanisms of jute-FRGC (2% wt%); (**c**) Fiber-matrix bonding in sisal-FRGC (3% wt%); (**d**) Failure mechanisms of sisal-FRGC (3% wt%) [63].

In addition to macroscopic evaluations, microscopic techniques provide critical insight into the interfacial behavior between plant fibers and the geopolymer matrix. High-resolution SEM and EDS analyses have been used extensively to visualize fiber-matrix bonding states, crack propagation patterns, and elemental distributions such as Na, Al, and Si [63,65]. SEM investigations indicate that sisal fibers possess rough, grooved surfaces that facilitate mechanical interlocking, whereas jute fibers exhibit smoother surfaces with less interfacial friction [Figure 5].

Similar to cementitious materials, the interfacial stress transmission between the fibers and the matrix in FRGPC, along with the composite’s behavior in the post-peak area, is regulated by three principal mechanisms: (i) Chemical connection between the fibers and the matrix (adhesion), (ii) friction produced when the fibers are extracted from the matrix and traverse inside the channel, and (iii) mechanical resistance offered by the fiber anchorage (hooked or deformed geometry of the fibers) [45,65].

Advanced methods such as synchrotron X-ray tomography and digital image correlation (DIC) allow non-destructive 3D interface reconstruction and real-time strain mapping during loading [66,67], providing dynamic insights into damage evolution. At the nanoscale, atomic force microscopy (AFM) and molecular dynamics (MD) simulations provide quantitative evidence of interfacial adhesion and predict mechanisms of bond formation [68,69,70,71].

Notwithstanding their benefits, these procedures exhibit disadvantages, such as elevated costs, intricate sample preparation, and an absence of standardized testing protocols. Consequently, additional research is required to develop dependable multiscale evaluation frameworks and in situ methodologies for assessing interfacial performance under prolonged service circumstances.

## 3. Mechanical Properties of Plant Fiber-Reinforced Geopolymer Concrete

### 3.1. Compressive Strength

Compressive strength is a crucial measure of the mechanical properties of concrete materials. The effect of fiber admixture on compressive strength is complex, principally determined by elements like fiber type, admixture content, and mixture ratio. The integration of plant fibers, when used in appropriate quantities, has a minimal effect on the compressive strength of geopolymer concrete, potentially resulting in a tiny reduction in certain cases.

The development of compressive strength in plant fiber-reinforced geopolymer concretes significantly differs from that of conventional cementitious composites. The inclusion of fibers at reduced doses (≤1.0 wt%) has demonstrated an enhancement in compressive strength attributed to better crack resistance and densification effects. In contrast, high dosages (≥1.0 wt%) sometimes lead to a decrease in strength, a fact ascribed to fiber aggregation and heightened matrix porosity [72,73,74].

It is important to note that the optimal fiber content varies considerably depending on the type of fiber and the characteristics of the matrix. Studies by Alomayri et al. [72] and Korniejenko et al. [74] demonstrated that moderate incorporation (around 0.5–1.0 wt%) of cotton, coconut, and ramie fibers provided notable strength improvement. Conversely, higher levels of incorporation, particularly with bamboo, raffia, and sweet sorghum fibers, have been observed to result in adverse effects, including fiber agglomeration and compromised matrix continuity [44,73,75]. Furthermore, as reported by Ayeni et al. [26], the incorporation of red hemp, coconut, and oil palm fibers beyond a certain threshold consistently led to a decline in compressive performance owing to structural discontinuities and enhanced porosity.

A summary of specific optimal dosages, fiber types, and associated quantitative results is presented in Table 2.

The compressive strength of PFRGC demonstrated considerable variety across many experiments, ranging from 20 to 89 MPa. A crucial doping level, generally between 0.5% and 1.0%, has been recognized as a significant determinant affecting the compressive strength of PFRGC. Below this crucial threshold, the addition of a small quantity of fibers has been shown to strengthen the matrix structure, decrease porosity, and boost densification, thereby slightly increasing the compressive strength. Conversely, beyond the critical threshold, the compressive strength generally diminishes, primarily due to the following factors: (1) the strength and stiffness of plant fibers are inferior to that of the GPC matrix, and an excessive quantity of fibers reduces the matrix material’s proportion; (2) plant fibers exhibit high absorbency, which interferes with the hydration reaction and hardening process; and (3) a substantial quantity of fibers tends to agglomerate within the matrix, creating defects and compromising the material’s homogeneity and compactness.

### 3.2. Tensile and Flexural Strength

**Figure 6:**
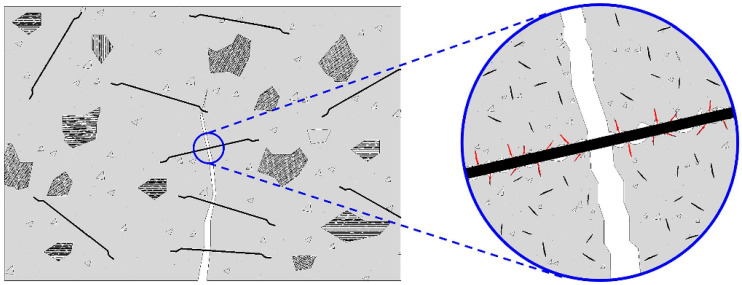
Mechanism of whisker enhancement for macroscopic fiber pull-out friction [77].

Unlike compressive strength, plant fibers provide a significant enhancement to the tensile and flexural strength of geopolymer concrete. Essentially a brittle material with high compressive strength but low tensile strength, plant fibers, under their high tensile strength and elongation, can take up tensile stresses when PFRGC is subjected to tensile or flexural stresses, and effectively inhibit crack extension [Figure 6].

The integration of plant fibers has repeatedly demonstrated enhancements in the tensile and flexural properties of geopolymer composites, especially when utilized at moderate levels. Table 3 indicates that fibers including sweet sorghum, pineapple, coconut, and bamboo demonstrate considerable strengthening effects, with optimal enhancements often attained within the range of 0.5–2.0 wt% or vol%. For instance, sweet sorghum and pineapple fibers have demonstrated strength enhancements of up to 36% and nearly 40%, respectively [75,78], while alkali-treated bamboo fibers have been reported to improve flexural strength by more than twofold [79].

However, the advantages of fiber supplementation are not infinite. Excessive fiber content can lead to issues such as inadequate dispersion, fiber aggregation, and heightened porosity, which eventually compromise the mechanical integrity of the matrix [80]. This restriction is notably apparent in research utilizing flax fibers at elevated doses, where anomalously high strength enhancements, such as 1107%, have been documented, yet remain contentious due to concerns about their experimental reproducibility [81].

The reinforcing efficacy of plant fibers is determined not only by their quantity but also by inherent characteristics such as aspect ratio, surface roughness, and chemical compatibility with the binder. The inherently coarse surfaces of bamboo fibers and the enhanced interfacial bonding resulting from alkali treatment of pseudo-banana fibers are pivotal in crack bridging and energy dissipation during tensile and flexural stress [79,82].

Taken together, these findings suggest that maximizing the mechanical performance of plant fiber-reinforced geopolymers requires careful control of fiber dosage, fiber morphology, dispersion uniformity, and interfacial engineering-elements that warrant further attention in both future experimental studies and practical applications.

**Table 3:** Summary of tensile and flexural strength enhancements in PFRGCs with various plant fiber types and dosages.

Fiber Type	Matrix	Fiber Dosage	Tensile Strength Enhancement	Flexural Strength Enhancement	Remarks	References
Sweet Sorghum	FA-based	2 wt%	↑ 36%	↑ 51%	Optimal at 2 wt%, decline at 3 wt%	[75]
Cotton	FA-based	0–4.1 wt% (Pre-dried)	-	↑ 50% (8 → 12 MPa)	Optimal at 2.1 wt%, decline at 2.8 wt%	[83]
Cotton	FA-based	0–1 wt%	-	↑ 12.5% (10.4 → 11.7 MPa)	Optimal at 0.5 wt%, decline beyond 0.5 wt%	[84]
Pineapple	FA-based	0–0.5 wt% (10–30 mm length)	-	↑ from 6.58 MPa to 9.21 MPa	Optimal at 0.5 wt%, dosage at 30 mm	[78]
Sisal	FA-based	0–1 vol%	↑ 73.6%	↑ 112.9% (3.1 → 6.5 MPa)	Increased strength with content up to 1 vol%	[85]
Coconut	FA-based	0–1 vol%	↑ 15.8%	↑ 106.5% (3.1 → 6.4 MPa)	Increased strength with content up to 1 vol%	[85]
Bamboo	MK-based	1–5 vol%	-	↑ 151% (3.9 → 9.8 MPa)	Optimal enhancement observed at 3 vol%	[79]
Pseudo-banana	FA-based	0.7–2 vol% (5–20 mm length)	↑ 33%	-	Optimal at 1.4 vol%, dosage at 10 mm	[82]
Coconut	FA-based	0–1 vol%	↑ 34.5%	↑ 47.0% (4.11 → 6.04 MPa)	Optimal at 0.75 vol% for flexural	[26]
Kenaf	FA-based	0–1 vol%	↑ 50%	↑ 59.7% (4.93 → 7.87 MPa)	Optimal at 0.75 vol% for flexural	[26]
Oil Palm	FA-based	0–1 vol%	↑ 28.5%	↑ 45.5% (4.02 → 5.85 MPa)	Optimal at 0.75 vol% for flexural	[26]
Flax (questionable)	Halloysite	0–10 wt%	-	↑ 1107% (5.8 → 70.2 MPa)	Results contested	[81]

Note: wt% = weight percentage; vol% = volume percentage; ↑ = increase; → = change from one value to another.

### 3.3. Toughness and Ductility

Toughness and ductility are critical indicators for evaluating the deformability and energy absorption properties of concrete materials. Plant fiber-reinforced geopolymer concrete (PFRGC) has demonstrated notable advantages in enhancing toughness and ductility.

The enhancement of toughness and ductility in GPC by plant fibers is primarily attributed to their role in bridging and crack control. When cracks emerge in the matrix, the fibers assume the tensile stresses by traversing the cracks, impeding their propagation. Concurrently, the fibers absorb a substantial amount of energy during tensile withdrawal or fracture. Moreover, the incorporation of plant fibers can encourage the refinement and dispersion of cracks, thereby enhancing the material’s overall deformation adaptability [86,87].

However, there were significant differences in the enhancement effect of different types of plant fibers on the toughness and ductility of GPC. Chen et al. [75] demonstrated that the post-peak toughness under split tensile loading increased by 1100%, 1600%, and 700%, respectively, when the fly ash-based polymer was reinforced with sweet sorghum fibers (doped with 1%, 2%, and 3% of the mass of the fly ash). A moderate amount of fiber incorporation resulted in a significant improvement in toughness; however, excessive incorporation led to a reduction in this improvement. In a similar study, Alomayri et al. [84] found that the toughness of 10 mm-long cotton fibers reinforced with fly ash-based polymer slurry at 0.5 wt% doping increased by approximately 60% in a three-point bending test. However, an increase in fiber doping to 0.7 wt% and 1.0 wt% resulted in a decrease in toughness due to a reduction in fiber dispersion.

Conversely, hemp fibers have been demonstrated to enhance GPC ductility and energy absorption. Research conducted by Poletanovic et al. [88] substantiated this finding. The energy absorption capacity of the geopolymer mortar was augmented by approximately 163% with the integration of 1 vol% of hemp fibers (10 mm in length). Following NaOH surface modification, the fiber-matrix interfacial bonding was strengthened, thereby augmenting the energy absorption capacity by approximately 13%. The hemp fiber-reinforced system demonstrates resilience in withstanding high residual loads in a cracked state, thereby enhancing the ductility of the material.

An appropriate increase in fiber doping has been shown to enhance toughness and ductility. However, excessive doping can lead to adverse effects on workability and strength. Furthermore, the contribution of different plant fibers to toughness and ductility varies due to differences in their tensile properties and modulus of elasticity. In general, the selection of plant fibers with high elongation and moderate modulus of elasticity is more conducive to the improvement of ductility [17]. The uniform dispersion and proper orientation of the fibers (particularly perpendicular to the crack propagation direction) can enhance the deformation and energy absorption properties of the material. The uniform dispersion and proper orientation of the fibers (particularly perpendicular to the direction of crack propagation) can optimize their bridging role and further improve the deformation and energy absorption properties of the material [26].

### 3.4. Impact Resistance Properties

Impact loading, a significant form of dynamic loading, is a common occurrence in the service life of building structures, including but not limited to earthquakes, explosions, impacts, and shocks. The micro-mechanism of the enhanced impact resistance of plant fiber-reinforced GPC can be primarily attributed to three factors: energy absorption, crack control, and stress redistribution. Under the impact load, the plant fiber absorbs the impact energy through its deformation and relative slip at the interface with the matrix. The bridging effect of the fiber effectively inhibits the rapid expansion of cracks and changes the state of stress distribution, thus improving the overall impact resistance of the material [89,90].

In the presence of external forces acting upon a fiber-reinforced geopolymer specimen, a complex stress field is generated internally. The distribution of fibers is of particular significance, as they form a three-dimensional network structure that functions as a bridge during the initiation and expansion of cracks. This results in a substantial increase in the material’s energy absorption capacity. Ige Samuel Ayeni et al. [26] The outcomes of the drop impact test (φ80 × 160 mm cylindrical specimen, 4.5 kg drop, 1 mm fall height) based on the ACI 544 (2004) standard demonstrated a substantial enhancement effect of the plant fiber on the impact resistance of GPC. The experimental data indicate a nonlinear relationship between the fiber volume fraction and the impact resistance performance, exhibiting an evident critical volume effect. When the fiber volume percentage was elevated from 0% to 0.75%, the impact strength of specimens reinforced with kenaf, coconut, and oil palm fibers improved by 11.35%, 3.77%, and 2.09%, respectively. Moreover, when the fiber content was elevated to 1.0%, the impact strength showed a pronounced nonlinear enhancement, increasing by 122.11%, 98.33%, and 88.33%, respectively.

The mechanism of this critical volume effect can be attributed to the formation threshold of the fiber three-dimensional network structure. When the fiber content is lower than the critical value, the fiber distribution is relatively dispersed, and it is difficult to form an effective spatial network structure. Conversely, when the fiber content reaches or exceeds the critical value, the fibers begin to form a continuous three-dimensional network, which significantly improves the material’s ability to absorb and disperse impact energy. Among the three natural fibers, kenaf fiber exhibits the most pronounced impact enhancement effect, which can be primarily ascribed to its high tensile strength (400–800 MPa) and exceptional interfacial bonding properties.

The Zwick Charpy Impact Tester is a tool used to assess the micro-impact resistance of materials through testing specimens [91]. The Charpy impact strength (*σ_i_*) is calculated by the ratio of the impact energy (*E*) required to destroy the sample to the sample fracture area (*A*):

σi=E/A

The impact strength of fiber-reinforced geopolymer composites is determined by three key factors: the quality of the fiber-matrix interfacial bond, the mechanical properties of the matrix, and the inherent qualities of the fibers [86]. Alomayri et al. [83] A systematic examination was conducted to ascertain the effect of cotton fiber content on the impact strength of geopolymer composites Charpy. The experimental results demonstrated a significant increase in impact strength with an increase in cotton fiber content in the dry composites. Specifically, the addition of 4.5, 6.2, and 8.3 wt% of cotton fibers increased the impact strength from 1.9 kJ/m^2^ to 6.2, 8.5, and 13.4 kJ/m^2^, respectively, with a maximum enhancement of 605%. This significant enhancement effect is primarily attributed to the increased elongation at break due to the high cellulose content of cotton fibers (approximately 88–96%), which increases the overall elongation at break of the composites and, consequently, the impact strength.

The researchers determined through a comprehensive fracture morphology investigation that, under sudden impact loading, composites lose energy via three principal mechanisms: fiber pullout, fiber fracture, and matrix deformation [92]. The relative contributions of these three mechanisms are contingent upon the strength of the fiber-matrix interfacial bond, the mechanical properties of the fibers themselves, and the deformability of the matrix.

A notable distinction emerges in the impact resistance of GPC when exposed to diverse categories of fibers. In addition to plant fibers, studies that investigate steel fiber reinforcement in GPC offer substantial insights. Rayapudi’s study [93] demonstrated that optimized 2% steel fiber-reinforced geopolymer concrete exhibited a 30.19% increase in compressive strength, a 36.67% improvement in splitting tensile strength, and a 75.87% rise in flexural strength, as compared to plain GPC. In GPC slabs with 0.5% to 2% steel fiber content, the initial cracking load increased by 1.25–2.5 times compared to ordinary GPC slabs. These data establish a standard for evaluating the performance of plant fibers in comparison to other types of fibers.

Hybrid fiber systems demonstrate superior impact resistance. Optimal blends of coir and polypropylene fibers are achieved at a total content of 0.3%, exhibiting impact resistance that is up to twice that of single-fiber concrete and up to three times that of standard concrete [94]. This synergistic reinforcement effect is derived from the complementary roles of different types of fibers in terms of scale, stiffness, and interfacial properties. The more rigid fibers primarily control macroscopic crack propagation, while the flexible fibers effectively inhibit microscopic crack sprouting. Collectively, these fibers enhance the overall impact resistance of the material. This finding provides a theoretical foundation for the design of multiscale fiber reinforcement.

In summary, the incorporation of plant fiber reinforcement demonstrated a substantial enhancement in the impact resistance of geopolymer concrete. Through the optimization of fiber type, content, and distribution, the material exhibited an impact strength enhancement ranging from 88% to 605%, accompanied by an increase in the number of first cracking impacts by 1.25 to 2.5 times. The study revealed that distinct fibers exhibited varied reinforcing effects, underscoring the necessity for identifying the optimal fiber content to ensure optimal performance [95].

## 4. Durability of Plant Fiber-Reinforced Geopolymer Concrete

Durability is a critical index for evaluating the long-term service performance of building materials. The effect of plant fiber incorporation on the durability of GPC is complex and multifaceted, with both positive and possible negative effects. Upon the complete condensation of hydration products, a stabilization of the pH level occurs [96]. This phenomenon is attributed to the provision of conducive conditions that facilitate the durability of plant fibers within the matrix.

### 4.1. Freeze-Thaw Resistance

The nature of freeze-thaw damage is a cumulative damage process in which the internal stress generated by the phase change and expansion of free water in the material during freezing at low temperatures exceeds the tensile strength threshold of the material, resulting in the development and proliferation of a network of microcracks. In the composition of geopolymers, water is generally present in three states: crystalline water, gel water, and free water [97]. Only the free water within the capillary pore network will crystallize and experience volume expansion as the temperature falls below the freezing point. This expansion is the primary catalyst for frost heave damage.

The influence of plant fibers on the freeze-thaw characteristics of geopolymer composites exhibits a distinct duality. In the low dosage range (volume fraction < 2%), the fibers mainly inhibit the initiation and propagation of microcracks during freeze-thaw cycles through a three-dimensionally distributed bridging network [98], and dissipate part of the frost heave stress through interfacial slip and pull-out processes [99], thereby significantly improving the material’s resistance to frost heave damage. Ekiz Barış and Tanaçan’s [100] experiments confirmed that the strength retention of a geopolymer with 1.5% banana fiber was approximately 40% superior to that of the control following 300 rigorous freeze-thaw cycles. The microscopic mechanism of this enhancement effect is that the spatial network structure of the fibers creates stress transfer channels, blocking the continuous propagation of cracks during freeze–thaw cycles.

Nevertheless, when the fiber content surpasses a crucial threshold, the freeze-thaw performance diminishes markedly. The internal mechanism of this phenomenon can be attributed to three key factors: Initially, an abundance of fibers results in heightened water absorption of the material, hence augmenting the quantity and distribution range of freezeable water. Second, the agglomeration effect caused by high fiber content destroys the continuity of the matrix and forms high-porosity areas. In particular, the proportion of macropores with a diameter exceeding 200 nm increases, these pores are more likely to undergo destructive expansion during the freezing process; third, the plant fiber experiences structural breakdown due to the combined influence of an alkaline environment and freeze-thaw cycles, resulting in diminished mechanical capabilities and a reduction in the reinforcing effect [101]. Tan et al. [102] systematically investigated the quantitative relationship between the content of rice straw fibers and freeze-thaw damage, determining that 8% represents the ideal fiber content, resulting in the minimal mass loss rate of 0.31% after 50 freeze-thaw cycles. Upon increasing the fiber content to over 12%, the mass loss rate of the material increased sharply to above 0.82%, indicating a significant reduction in freeze-thaw resistance.

**Figure 7:**
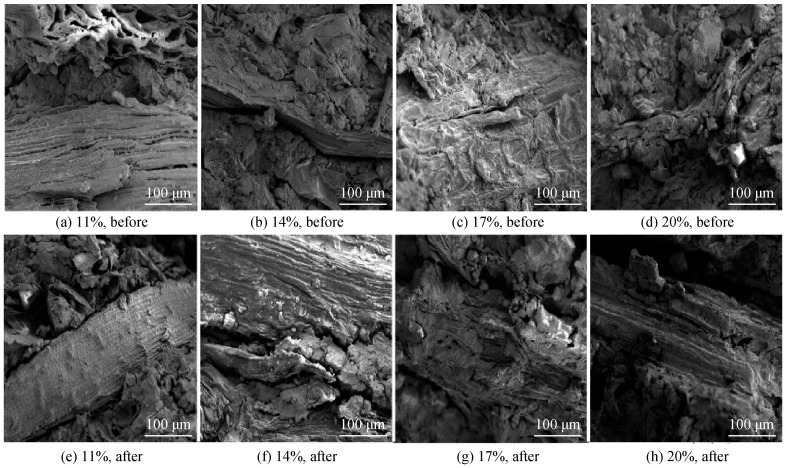
SEM images of geopolymer composites with different fiber content before and after freeze-thaw [103].

Research by Xuan et al. [103] offers significant insights into the correlation between the microstructure of plant fiber-reinforced GPC and its resistance to freeze-thaw cycles. After 25 freeze-thaw cycles, the overall integrity was maintained, with a mass change rate of only 0.1% to 4%, and no macroscopic damage such as surface collapse, missing corners, or obvious cracks. Microscopic investigation revealed that freeze-thaw cycles resulted in the loosening of the matrix structure surrounding the fibers, leading to the emergence of microcracks at the fiber-matrix interface [Figure 7].

Through the examination of the progression of the fiber-matrix interface in a freeze-thaw environment, two technical approaches may be identified to enhance the freeze-thaw performance of plant fibers reinforced with GPC: Hydrophobic alteration of the fiber surface can decrease the water absorption rate and expansion propensity of the fiber, hence mitigating interfacial stress during the freeze-thaw process [104]; on the other hand, The incorporation of active fillers like fly ash can enhance the pore structure of the material, facilitate the development of N-A-S-H gel, occupy the tiny gaps around the fibers, and augment the interfacial bond strength [101]. This fiber-fly ash synergistic system can retain 85–90% of its strength after 100 freeze-thaw cycles, which is significantly better than a single-component system.

The freeze-thaw performance of GPC reinforced with plant fibers demonstrates considerable environmental dependence. Experimental data analysis shows that under moderate freeze-thaw conditions, the bridging of cracks by fibers dominates, and the material exhibits excellent freeze-thaw resistance; however, under extreme freeze-thaw conditions, fiber degradation and interfacial damage accelerate, and the freeze-thaw performance may decrease sharply.

### 4.2. Permeability Resistance

Permeability resistance is a critical index for evaluating the capacity of concrete to impede the infiltration of water and deleterious substances. It directly correlates with the material’s long-term durability in wet and erosive environments. The primary mechanism by which plant fibers enhance the seepage resistance of GPC encompasses two aspects: pore structure regulation and crack control. Adequate fiber incorporation can optimize the pore structure, reduce the proportion of large pores, and limit pore connectivity. Concurrently, the three-dimensional network structure of fibers can effectively impede the creation and propagation of microcracks and diminish the occurrence of through cracks via the bridging effect, thereby reducing the connectivity of infiltration channels.

The hydrophilicity of plant fibers exerts a substantial influence on the impermeability of GPC. Nkwaju et al. [105] ascertained that the water absorption of bagasse fiber-reinforced geopolymer composites exhibited a substantial increase following dry and wet cycling, a phenomenon that was predominantly ascribed to the intrinsic hydrophilicity characteristic of plant fibers. The water-absorbing characteristics of diverse plant fibers exhibited substantial variation. Specifically, ramie fiber-reinforced geopolymers demonstrated the most pronounced increase in water absorption, ranging from 17% to 29% higher than that of the unreinforced samples. Conversely, hemp fiber-reinforced samples had the minimal increase in water absorption, attaining just around 3% more than the unreinforced samples. This mismatch is due to the varying levels of hemicellulose, lignin, and cellulose, which directly affect the hydrophilicity and pore structure of the fibers.

Fiber surface modification treatment has been demonstrated for enhancing fiber-matrix interfacial adhesion and enhancing the impermeability of GPC. Poletanovic et al. [44] have demonstrated that the 9% NaOH-treated hemp fibers exhibited a reduction in fiber absorption of water of approximately 20% and a decrease in mortar porosity of around 26%. This substantial improvement in impermeability was observed in fly ash-based alkali-activated mortar following wet and dry cycling. The alkali treatment has been shown to reduce fiber hydrophilicity and enhance fiber-matrix interfacial bonding by eliminating hemicellulose, pectin, and waxy substances from the fiber surface. In contrast, untreated fibers have been observed to potentially diminish impermeability due to their high water absorption and interfacial defects.

In the context of dry and wet cycling conditions, the bridging effect of plant fibers has been demonstrated to effectively impede microcrack extension and thereby enhance material durability. Yanou et al. [106] observed that geopolymer composites incorporating 3 wt% of bagasse fibers exhibited significantly diminished mass loss and strength reduction compared to unreinforced samples after 20 cycles of dry and wet cycling. Santos et al. [107] research has demonstrated that sisal fiber-reinforced alkali-activated cements demonstrate commendable durability, exhibiting up to 73% flexural strength retention after 10 wet and dry cycles.

The impact of plant fibers on the anti-chlorine ion permeation properties of GPC exhibited clear non-linear characteristics, suggesting the existence of an optimal combination of fiber parameters. The addition of 0.75% volume fraction of kenaf fiber into GPC significantly enhanced its anti-chlorine ion permeation properties. After a 90-day immersion period, the depth of chlorine ion permeation through composites with fibers measuring 20 mm, 30 mm, and 40 mm was reduced by 20.9%, 25%, and 16.36%, respectively. The influence of fiber length on the impermeable performance exhibited clear threshold characteristics. Specifically, the impermeable performance reached its peak at a fiber length of 30 mm, and further increases in fiber length, up to 40 mm, resulted in a 23.7% increase in penetration [108]. This trend followed a typical “first decline and then rise” pattern. This phenomenon is primarily attributable to the propensity of excessively elongated fibers to coalesce, resulting in an augmentation of local porosity and the emergence of infiltration channels.

The combined effect of plant fibers and mineral admixtures has been shown to enhance the seepage resistance of GPC. As Wei et al. [109] have found, the combination of cellulose fibers and fly ash has been demonstrated to enhance the durability of composites under dry and wet cycles of sulfate. Furthermore, the ratio optimization enhances the prevalence of benign pores, including micropores and tiny pores [110]. This synergistic effect is derived from the involvement of fly ash in the geopolymerization reaction, leading to the creation of a gel product that fills a portion of the pores. The fibers simultaneously obstruct the advancement of microcracks.

Nonetheless, the influence of plant fibers on the impermeability of GPC is not consistently advantageous. Gulzar et al. [111] assert, the chloride penetration depth of concrete in a 10% NaCl solution exhibited a 27% increase with the incorporation of 0.5% jute fibers, primarily due to the increased porosity of the composite material resulting from the jute fibers. Nevertheless, this adverse effect can be efficiently mitigated by the addition of high-efficiency water-reducing additives, thereby reducing the depth of chloride ion penetration by approximately 17%. The reduction of the liquid-solid ratio has been shown to effectively diminish the porosity of the composite material, consequently enhancing its permeability resistance [112].

Incorporating sufficient fiber quantities, appropriate fiber lengths, homogeneous distribution, and surface modification has demonstrated that plant fibers improve the resistance to water and chloride ion penetration, as well as the durability of GPC in both wet and dry environments. Conversely, in adverse conditions marked by high fiber content, excessively long fibers, uneven distribution, and untreated hydrophilic fibers, penetration resistance may diminish.

### 4.3. Carbonation Resistance

Carbonation represents a primary concern in terms of durability for concrete structures during their service life. This phenomenon is the result of complex physicochemical interactions between atmospheric CO_2_ and the binder matrix. In conventional Portland cement concrete, carbonation mostly transpires via the reaction between Ca(OH)_2_ and the calcium silicate hydrate (C-S-H) gel with CO_2_, resulting in the formation of calcium carbonate (CaCO_3_). The carbonation mechanism in geopolymer concrete (GPC) is inherently distinct. In GPC, the process of carbonation primarily entails the interaction of CO_2_ with N-A-S-H and C-(A)-S-H gels in the pore solution, leading to the formation of carbonate and bicarbonate compounds, such as Na_2_CO_3_ and NaHCO_3_ [113,114]. This process leads to a decrease in the pH of the pore solution and induces gel structure rearrangement and changes in the distribution of pore size. While GPC typically exhibits greater carbonation depth than ordinary Portland cement concrete [115]. The influence of plant fibers on the carbonation resistance of GPC has been minimally addressed in the existing research literature.

Huang et al. [116] conducted a comparative study that revealed that environmental conditions had a substantial impact on the carbonation process of plant fiber-reinforced GPC. The outdoor-exposed samples exhibited the highest degree of carbonization, followed by the indoor samples, while the hermetically stored samples showed the lowest degree of carbonization. This result suggests that ambient humidity and CO_2_ concentration are the key factors affecting the carbonation process. The study demonstrated that carbonization has a dual impact on the matrix’s durability and the interfacial binding fidelity among the plant fibers and the matrix, hence influencing the composite’s overall performance. The external environment inflicted the most significant harm to the geopolymer matrix, plant fibers, and their interfacial adhesion, whereas the fiber strength of the hermetically sealed samples diminished despite the matrix’s relative integrity. This finding emphasizes the decisive role of the stability of the interfacial adhesion between plant fibers and the geopolymer matrix regarding anti-carbonation capabilities. Ildiko Merta et al. [117] Systematic studies under accelerated carbonation conditions demonstrated that hemp fibers promoted the precipitation of alkaline and alkaline-earth metal salts in the carbonation reaction by increasing the initial porosity. This resulted in a significant reduction of mortar water uptake (~20%), with a particularly pronounced effect in the fly ash (FA) matrix. The enhancement of the pore structure is advantageous for the material’s long-term durability. The carbonation-induced embrittlement of the matrix and fiber/matrix interface compromised the reinforcement provided by the fibers, demonstrating a distinct dual impact. This effect manifested distinctly across diverse matrix systems: the FA-based mortar demonstrated an improvement of around 40% in compressive strength and 34% in flexural strength attributable to the sufficient carbonation reaction. The embrittlement process resulted in a 7% decrease in energy absorption capability. Conversely, the FA/GBFS (blast furnace slag)-based mortar demonstrated a minimal variation in strength (±10%), due to the decalcification of the high-calcium system and the limited infiltration of CO_2_. Furthermore, the elevated fiber dosage, the research indicated that a slight augmentation in fiber content (0.5 vol%) can result in a significant reduction in strength (14–23%). This observation highlights the crucial importance of fiber content in enhancing material characteristics. The results demonstrate that a fiber dose of 0.5 vol% is best for achieving a balance of durability, strength, and energy absorption capability.

The impact of carbonation on the pH of the GPC pore solution is a critical indicator for evaluating the resistance to carbonation. Pouhet and Cyr [118] found that the pH of the pore solution in the geopolymer matrix derived from metakaolin cured in a natural CO_2_ environment for 365 days decreased to a lesser extent than that of the ordinary cement matrix, stabilizing at around 10.5. This evidence indicates that GPC can still maintain a certain alkaline environment during long-term carbonation, which is conducive to maintaining the stability of the material. The leaching behavior of sodium carbonate (Na_2_CO_3_), the main product of carbonation, as a water-soluble substance, exerts a significant impact on the long-term efficacy of the geopolymer.

The beneficial impact of plant fibers on the carbonation resistance of GPC can be attributed to two primary factors. Initially, the three-dimensional network configuration of the fibers retards the rate of carbonation by enhancing microstructural integrity and impeding CO_2_ infiltration [119]. Secondly, the crack-mitigating effect of fibers may diminish the initiation and propagation of microcracks during carbonation, hence preserving the structural integrity of the material. In addition, a moderate amount of plant fibers may improve the carbonation resistance through the optimization of the pore architecture of GPC, increasing the material compactness, and reducing the permeability of CO_2_. However, under unfavorable conditions (excessive fibers, poor interfacial bonding, harsh environmental conditions), the fibers may increase the material porosity, accelerate the carbonation process, and even lead to performance degradation due to interfacial embrittlement.

### 4.4. Chemical Resistance

Chemical corrosion resistance is a critical metric for assessing the long-term durability of concrete materials under specific conditions. Under the action of acid rain, sewage, marine environments and other aggressive media, the concrete structure is prone to chemical corrosion, resulting in material expansion and cracking, performance deterioration and even structural damage. Geopolymer concrete (GPC) itself usually has superior chemical corrosion resistance to ordinary silicate cement concrete [120] and the impact of integrating plant fibers on the chemical corrosion resistance of GPC has a complex dual nature.

A systematic investigation of the durability of hemp fiber-reinforced foam concrete in 5% magnesium sulfate (MgSO_4_) and 5% sodium sulfate (Na_2_SO_4_) solutions by Osman Gence [101], the results revealed the complex interaction between plant fibers and mineral admixtures in different sulfate environments. In the magnesium sulfate environment, hemp fiber concrete with high fly ash content (50%) performed best with a strength loss of only 4.7%, while concrete with 30% fly ash admixture had the highest loss (23%); in the sodium sulfate environment, hemp fiber concrete with 10% fly ash admixture performed best with a strength loss of only 4.8%. This disparity signifies the ideal proportion of plant fibers to mineral admixtures is highly dependent on the type of erosion medium. In addition, hemp fiber-reinforced concrete showed better sulfate resistance with minimal loss of flexural strength (9%) at low foam content (50 kg/m^3^), which was mainly attributed to lower porosity and higher material compactness. The mechanism is mainly reflected in two aspects: on the one hand, the fibers effectively inhibit the microcrack extension caused by sulfate erosion through bridging, maintaining the structural integrity of the material; on the other hand, the hydrophilicity of the fibers may lead to the absorption of sulfate solution in localized areas and the formation of microcrack origination points under long-term erosion. Mass loss analysis showed that high fly ash content (50%) significantly reduced the solubility loss due to sulfate erosion, while the incorporation of hemp fibers had a very minor impact on mass loss, suggesting that the plant fibers improved the sulfate resistance mainly by influencing the microstructure and crack control of the material.

The nonlinear nature of the influence of plant fibers on the sulfate resistance of GPC was further confirmed by Abbas et al. [108]. The PFRGC exhibited satisfactory sulfate resistance when a moderate amount of kenaf fiber (0.75–1.0%) and short fiber (20–30 mm) was incorporated. However, when the fiber doping was further augmented, the sulfate resistance exhibited a decline due to the escalating porosity resulting from fiber agglomeration. This behavior was ascribed to the moderate quantity of fibers creating a lattice structure inside the geopolymer, thus obstructing the ingress of deleterious particles into the matrix. In contrast, an overabundance of fibers resulted in an irregular distribution, impairing the adhesion of fibers to the matrix, elevating porosity, and diminishing durability. This discovery highlights the necessity of refining fiber properties to enhance the sulfate resistance of GPC.

Zhou et al. [121] discovered that cotton straw powder had a substantial negative impact on the acid resistance of alkali-activated materials (AAM). Following a 14-day exposure to a 2 M hydrochloric acid solution, the compressive strength of the reinforced composites was determined to be 13.5–23.5% diminished compared to that of the original composites. The reduction is mostly due to the high hydrophilicity and inadequate acid stability of the cotton straw powder employed in the composite. The addition of coconut husk fiber has been found to increase the matrix toughness and boost the composite’s resistance to acid corrosion [122,123]. This discrepancy is primarily attributable to the elevated lignin content and diminished hydrophilicity of coir fiber, characteristics that confer enhanced stability in acidic environments.

Sá Ribeiro et al. [124] study on kaolin-based polymers reinforced with bamboo fibers demonstrated that the integration of bamboo fibers improved the durability of geopolymer composites (GPC) in sulfuric and hydrochloric acid environments with a concentration of ≤15 wt%. The bamboo fibers exhibited a significant improvement in acid resistance through several mechanisms, including crack bridging, microcrack inhibition, and silica gel deposition. The mass loss of the geopolymer matrix rose from 2.7% to 4%. The concentration of sulfuric acid was elevated from 5 wt% to 15 wt%, yielding a 4% rise. The material showed a greater extent of strength deterioration in the H_2_SO_4_ environment than in the HCl environment. The research determined that bamboo fiber-reinforced geopolymer can be safely applied to building materials exposed to ≤15 wt% sulfuric or hydrochloric acid environments, including wastewater systems. However, at high acid concentrations or high fiber doping conditions, bamboo fibers may increase porosity and render the substrate more susceptible to corrosion.

### 4.5. Drying Shrinkage Behavior

The phenomenon of shrinkage in geopolymers can be classified into three distinct categories: chemical shrinkage, the phenomenon of auto-constriction has been observed, and drying shrinkage. In contrast to Ordinary Portland Cement In contrast to the conventional Portland cement, which undergoes three stages of chemical deformation: shrinkage, expansion, and subsequent shrinkage [125], drying shrinkage is indicative of the volumetric stability of the material during water loss and the potential risk of cracking. The integration of fibers diminishes the pressures exerted on the matrix, so effectively constraining its drying shrinkage.

The modulation of drying shrinkage properties of geopolymers by plant fibers demonstrates a substantial fiber type dependence. Systematic studies have demonstrated that under identical conditions, ramie, hemp, and bamboo fiber-reinforced geopolymers display diminished drying shrinkage, while jute, coir, and sisal fiber-reinforced geopolymers showed increased drying shrinkage compared to the unreinforced controls [44]. This mismatch can be ascribed to the increased surface roughness of jute, coir, and sisal fibers, which results in augmented porosity within the interfacial region and intensifies capillary water loss. Conversely, the mechanical confinement effect exhibited by ramie, hemp, and bamboo fibers supersedes the impact of their hygroscopic tendencies, thereby effectively curtailing matrix shrinkage.

Alcivar-Bastidas et al. [126] demonstrated that the alkali treatment of abaca fibers has demonstrated to reduce the 28-day drying shrinkage of geopolymer mortar by 13.5%. Moreover, the fiber-reinforced samples have demonstrated superior dimensional stability under wet-dry cycling conditions, thereby confirming the efficacy of the bridging effect of plant fibers in inhibiting microcrack extension. Compared to plant fibers, synthetic fibers like steel and PVA exhibit optimal doping thresholds for controlling geopolymer shrinkage at 1.0% and 0.3%, respectively, beyond which their efficacy reduces [51], the shrinkage exhibited a positive correlation with the water-cement ratio and the curing time.

The mechanism by which plant fibers affect the dry-shrinkage properties of geopolymers exhibits multiscale characteristics. At the microscopic level, Haily et al. found that bio-based fibers produced significantly fewer microscopic defects in the geopolymer matrix than synthetic fibers [127]. The hydrophilicity of plant fibers has a twofold impact on drying shrinkage characteristics. It improves overall hydrophilicity while intensifying drying shrinkage. Conversely, it serves as an internal conservation medium and prevents self-shrinkage [128,129]. This apparent contradiction reflects the complexity of plant fibers affecting drying and shrinkage, which necessitates precise control of fiber properties and matrix ratios to optimize the process.

The process of alkaline hydrolysis of cellulose and hemicellulose has been demonstrated to lead to a decrease in interfacial bond strength and an augmentation in drying shrinkage [130]. Xu et al. [131] has been confirmed that plant fibers can act as stress transfer and energy dissipation mechanisms to reduce crack extension. Maintenance conditions are also a key external factor influencing the dry shrinkage properties of plant fiber-reinforced geopolymers. Amran et al. [132] showed that the interplay between temperature and humidity exerts a synergistic impact on the drying and shrinkage properties of the material. At elevated temperatures, the curing process accelerates the development of strength in the material. However, this increase in strength is accompanied by an exacerbation of water evaporation and shrinkage. This phenomenon can be effectively mitigated by increasing the ambient humidity level to an appropriate extent.

## 5. Emerging Applications and Future Challenges of Plant Fiber-Reinforced Geopolymer

With the growing demand for green building materials, PFRGC is expanding into several innovative application areas. The introduction of renewable plant fibers into geopolymer systems is not only in line with the concept of a circular economy but also opens up new avenues for the development of lightweight, thermally insulating, and customizable building components. Walbrück et al. [133] researched the application of miscanthus fibers in geopolymer foams and found a strong bonding between the fibers and the matrix and an irregular pore distribution, which improves the thermal insulation properties. Under the optimal ratio, the thermal conductivity decreased by 55.1%. These properties make it a potential sustainable and environmentally friendly thermal insulation material suitable for walls and cooling systems. Similarly, Novais et al. [134] showed that cork-alkali-activated fly ash multifunctional building materials showed significant results in optimizing thermal conductivity (decreasing up to 67.6%) and excelled in thermal insulation, sound absorption and humidity regulation. In terms of fire resistance, Silva et al. [135] examined a fiber-reinforced lightweight pozzolana-based geopolymer that significantly improved thermal insulation and water absorption. In the fire resistance test, the material can absorb about 65% of the heat from the flame, the temperature on the back side is less than 350 °C, the surface is only slightly discolored, and the shape is stable. It has great application prospects.

Especially in the 3D printing of building materials, the addition of fibers can change the printability, shape retention and interlayer bonding strength of the geopolymer paste [136,137]. In recent years, researchers have attempted to introduce natural plant fibers into the geopolymer system to explore their potential for reinforcement and processing adaptability in 3D printed building materials. Kozub et al. [138] verified the feasibility of fly ash-based geopolymer in 3D printing and demonstrated the potential of cotton fibers to reduce thermal conductivity, but failed to improve mechanical strength. Kong et al. [139] showed that kenaf stalk and straw core can significantly optimize the rheological properties of geopolymer slurries, improve printability and extrusion continuity, while increasing shape retention (thickness retention rate of more than 90%) and interlayer bond strength (compressive strength of 8.39 MPa at 28 days for a three-layer sample). However, fiber agglomeration, increased porosity, and process optimization remain challenges.

Despite the considerable technical potential of PFRGC, several notable challenges remain in current research: (1) inadequate long-term stability of plant fibers in alkaline environments and the absence of effective degradation prevention methods; (2) difficulties in achieving uniform fiber dispersion, particularly at elevated fiber content; (3) a deficiency of quantitative models elucidating the interfacial bonding mechanisms between plant fibers and geopolymer; (4) obstacles in industrial production regarding quality consistency and cost management; and (5) a lack of standardized testing methodologies and design specifications, which constrains engineering applications. The performance deterioration of PFRGC under elevated temperature and humid conditions requires immediate attention.

## 6. Conclusions

This paper examines the current research related to the interfacial characteristics, mechanical behavior, and durability of plant fiber-reinforced geopolymer concrete and puts forward the following main conclusions based on the analysis:

This critical review employs a systematic literature evaluation method and conducts a comprehensive search of databases such as Web of Science and Science Direct (2000–2025) to provide a thorough understanding of the current state of plant fiber-reinforced geopolymer concrete (PFRGC). This work outlines the current state of research on PFRGC and identifies directions worthy of further investigation. The results suggest that the interfacial bonding between plant fibers and the geopolymer matrix is primarily governed by mechanical interlocking and chemical interactions. Notably, the interfacial transition zone exhibits a gradient structural morphology, reflecting the complexity of the fiber–matrix interface. Experimental evidence indicates that appropriate surface treatment of fibers (particularly via alkali conditioning and silane coupling) can effectively improve interfacial bonding strength. In addition, adjusting the Si/Al ratio and introducing nanomaterials contribute to the development of a more compact and chemically stable interfacial microstructure. The phase composition and morphology of the interfacial transition zone play a critical role in governing the macroscopic properties of fiber-reinforced geopolymer composites.

Quantitative studies indicate that the influence of plant fibers on the mechanical behavior of geopolymer concrete exhibits a nonlinear pattern. When added at a volume fraction between 0.5% and 1.5%, fibers have been shown to improve flexural strength by 20% to 45% and enhance toughness by 75% to 150%. Nonetheless, a modest decline in compressive strength (typically between 5% and 15%) is often observed. The toughening performance of plant fiber-reinforced geopolymer composites is strongly influenced by the fiber’s aspect ratio, geometry, surface chemistry, and dispersion quality within the matrix. These factors collectively determine the efficiency of stress transfer and crack-bridging mechanisms at the fiber–matrix interface.

Long-term durability studies reveal that plant fiber incorporation in geopolymer composites presents a trade-off between improved functional performance and material degradation risks. Properly integrated fibers enhance impermeability, chemical resistance, and dimensional stability by arresting crack propagation and redistributing internal stresses. However, their intrinsic hydrophilicity and chemical instability in alkaline environments can compromise the structural integrity of the composite over time. Addressing this issue requires a combined approach: modifying fiber surfaces to enhance chemical stability and tailoring the matrix composition to ensure interfacial compatibility and resistance to alkaline-induced deterioration.

PFRGC has exhibited considerable benefits in lightweight insulation materials, 3D printed construction, and specialized functional materials (fire resistance, sound absorption, moisture regulation), hence broadening its technological frontiers. Nonetheless, issues including regulated fiber dispersion, interfacial stability, and large-scale manufacturing must be resolved to fully actualize its potential in novel functional applications.

In summary, this paper provides an important scientific basis for understanding the interfacial mechanisms, performance optimization, and application potential of plant fiber-reinforced geopolymer concrete through literature analysis and critical evaluation. As interdisciplinary research continues to deepen, the application of plant fiber-reinforced geopolymer concrete is anticipated to expand to include large-scale implementation in the domains of green building, infrastructure maintenance, and special engineering. This development is expected to provide substantial technical support for the sustainable advancement of the construction industry.

## Figures and Tables

**Table 1:** Physical and mechanical characteristics of frequently documented plant fibers in literature.

Fiber Type	Fiber	Density (g/cm^3^)	Tensile Modulus (GPa)	Tensile Strength (MPa)	Elongation (%)	Organ Source Classification	Reference
Seed Fibers	Cotton	1.5–1.6	5.5–12.6	287–597	7.8–8.2	Seed epidermal unicellular fibers	[10]
Bast Fibers	Flax	1.4–1.5	27.6–103	343–2000	1.2–3.3	Primary phloem of flax stem	[10,11]
	Hemp	1.4–1.5	23.5–90	270–900	1–3.5	Cannabis sativa stem phloem	[10]
	Jute	1.3–1.49	8–78	320–800	1–1.8	Jute secondary phloem	[10]
	Ramie	1.0–1.55	24.5–128	400–1000	1.2–4.0	Ramie bast fiber bundle	[10]
	Kenaf	1.4	14.5–53	223–930	1.5–2.7	secondary phloem of red hemp (Jatropha curcas)	[10]
Leaf Fibers	Sisal	1.33–1.5	9.0–38	363–700	2.0–7.0	sisal leaf vascular fiber	[10]
	Abaca	1.5	6.2–20	400–980	1.0–10	Plantago lanceolata leaf sheath fiber bundle	[10]
	Pineapple leaves	0.8–1.6	144–825	180–1627	1.6–14.5	Pineapple Leaf Fiber	[10]
	Banana	1.35	12	500	1.5–9	Banana leaf sheath fiber bundle	[10]
Stalk Fibers	Bamboo	0.6–1.1	11–32	140–800	1.5–9	vascular fibers of bamboo stems	[10]
	Bagasse	1.25	17–27.1	222–290	1.1	Sugar cane stalk fiber	[10]
Fruit Fibers	Oil palm	0.7–1.55	0.5–3.2	80–248	17–25	Oil Palm Husk Fiber Bundle	[10]
	Coconut shell	0.8	3.5	174	25	Coconut fruit husk fiber bundle	[12]

**Table 2:** Summary of compressive strength results of PFRGCs with different fiber types and dosages.

Fiber Type	Matrix	Fiber Dosage	Length	Compressive Strength Trend	Remarks	Reference
Cotton	FA-based	0.5 wt%	10 mm	↑ 140%	Peak at 0.5 wt%, then ↓ at 0.7–1.0 wt%	[72]
Coconut, Cotton, Sisal, Raffia	FA-based	1.0 wt%	Cotton: 30 mm Others: 3 mm	Coconut ↑ 27%, Cotton ↑ 1 5%, Sisal ↑ 2%, Raffia ↓ 45%	Fiber type affects the trend	[74]
Sweet Sorghum	FA-based	1–3 wt%	≤50 mm	↓ 9–26%	Strength declines with increasing dosage	[75]
Bamboo	MK-based	5 wt%	≤40 mm	↓ 50%	Long fibers at high dosage are detrimental	[73]
Ramie, Coir, Sisal, Jute, Hemp	FA-based	1–2 wt%	≤25 mm	Ramie (1%) highest strength; others are slightly ↓	Ramie shows potential	[44]
Coconut Trunk	FA-based	0–1 g	30–50 mm	Peak at 0.5 g	Highest value 89.44 MPa	[76]
Kenaf, Coconut, Oil Palm	FA-based	0–1 vol%	25 mm	↓ With increasing fiber content	Due to increased porosity	[26]

Note: wt% = weight percentage; vol% = volume percentage; FA = fly ash; MK = metakaolin; ↑ = increase; ↓ = decrease.

## Data Availability

No new data were created or analyzed in this study. Data sharing is not applicable to this article.

## Abbreviations

The following abbreviations are used in this manuscript:

GPC	Geopolymer Concrete
PFRGC	Plant Fiber-Reinforced Geopolymer Concrete
FRGC	Fiber-Reinforced Geopolymer Concrete
AAM	Alkali-Activated Materials

## References

[1] Barbhuiya S., Kanavaris F., Das B.B., Idrees M. (2024). Decarbonising Cement and Concrete Production: Strategies, Challenges and Pathways for Sustainable Development. J. Build. Eng..

[2] International Energy Agency (2018). Low-Carbon Transition in the Cement Industry.

[3] Amran Y.H.M., Alyousef R., Alabduljabbar H., El-Zeadani M. (2020). Clean Production and Properties of Geopolymer Concrete; A Review. J. Clean. Prod..

[4] Unis Ahmed H., Mahmood L.J., Muhammad M.A., Faraj R.H., Qaidi S.M.A., Hamah Sor N., Mohammed A.S., Mohammed A.A. (2022). Geopolymer Concrete as a Cleaner Construction Material: An Overview on Materials and Structural Performances. Clean. Mater..

[5] Pham K.V.A., Nguyen T.K., Le T.A., Han S.W., Lee G., Lee K. (2019). Assessment of Performance of Fiber Reinforced Geopolymer Composites by Experiment and Simulation Analysis. Appl. Sci..

[6] Karimah A., Ridho M.R., Munawar S.S., Adi D.S., Ismadi, Damayanti R., Subiyanto B., Fatriasari W., Fudholi A. (2021). A Review on Natural Fibers for Development of Eco-Friendly Bio-Composite: Characteristics, and Utilizations. J. Mater. Res. Technol..

[7] Manso-Morato J., Hurtado-Alonso N., Revilla-Cuesta V., Skaf M., Ortega-López V. (2024). Fiber-Reinforced Concrete and Its Life Cycle Assessment: A Systematic Review. J. Build. Eng..

[8] De Azevedo A., Cruz A., Marvila M., De Oliveira L., Monteiro S., Vieira C., Fediuk R., Timokhin R., Vatin N., Daironas M. (2021). Natural Fibers as an Alternative to Synthetic Fibers in Reinforcement of Geopolymer Matrices: A Comparative Review. Polymers.

[9] Shalchian M.M., Arabani M. (2023). Application of Plant-Derived Fibers in Soil Reinforcement on Experimental, Numerical, and Case Study Scales: A Review. Bull. Eng. Geol. Environ..

[10] Dittenber D.B., GangaRao H.V.S. (2012). Critical Review of Recent Publications on Use of Natural Composites in Infrastructure. Compos. Part A Appl. Sci. Manuf..

[11] Bos H.L., Van Den Oever M.J.A., Peters O.C.J.J. (2002). Tensile and Compressive Properties of Flax Fibres for Natural Fibre Reinforced Composites. J. Mater. Sci..

[12] Tolêdo Filho R.D., Scrivener K., England G.L., Ghavami K. (2000). Durability of Alkali-Sensitive Sisal and Coconut Fibres in Cement Mortar Composites. Cem. Concr. Compos..

[13] Melo Filho J.D.A., Silva F.D.A., Toledo Filho R.D. (2013). Degradation Kinetics and Aging Mechanisms on Sisal Fiber Cement Composite Systems. Cem. Concr. Compos..

[14] Wei J., Meyer C. (2015). Degradation Mechanisms of Natural Fiber in the Matrix of Cement Composites. Cem. Concr. Res..

[15] Korniejenko K., Łach M., Hebdowska-Krupa M., Mikuła J. (2018). The Mechanical Properties of Flax and Hemp Fibres Reinforced Geopolymer Composites. IOP Conf. Ser. Mater. Sci. Eng..

[16] Ye H., Zhang Y., Yu Z., Mu J. (2018). Effects of Cellulose, Hemicellulose, and Lignin on the Morphology and Mechanical Properties of Metakaolin-Based Geopolymer. Constr. Build. Mater..

[17] Islam M.R., Ul Isalm M.R., Rashid M., Ahammad F., Islam M. (2024). Improvements in the Characteristics of Plant Fiber Reinforced Concrete. Eur. J. Theor. Appl. Sci..

[18] Cai M., Takagi H., Nakagaito A.N., Li Y., Waterhouse G.I.N. (2016). Effect of Alkali Treatment on Interfacial Bonding in Abaca Fiber-Reinforced Composites. Compos. Part A Appl. Sci. Manuf..

[19] Kumar R.S., Muralidharan N., Sathyamurthy R. (2022). Optimization of Alkali Treatment Process Parameters for Kenaf Fiber: Experiments Design. J. Nat. Fibers.

[20] Arsène M.-A., Okwo A., Bilba K., Soboyejo A.B.O., Soboyejo W.O. (2007). Chemically and Thermally Treated Vegetable Fibers for Reinforcement of Cement-Based Composites. Mater. Manuf. Process..

[21] Orue A., Jauregi A., Unsuain U., Labidi J., Eceiza A., Arbelaiz A. (2016). The Effect of Alkaline and Silane Treatments on Mechanical Properties and Breakage of Sisal Fibers and Poly(Lactic Acid)/Sisal Fiber Composites. Compos. Part A Appl. Sci. Manuf..

[22] Majhi S., Pradhan S., Prakash V., Acharya S.K. (2023). Influence of Alkali Treatment on the Interfacial Shear Strength of *Agave lechuguilla* Fiber and Its Significance as a Reinforcing Material in Polymer Composites for Mechanical Applications. Polym. Compos..

[23] Oushabi A., Sair S., Oudrhiri Hassani F., Abboud Y., Tanane O., El Bouari A. (2017). The Effect of Alkali Treatment on Mechanical, Morphological and Thermal Properties of Date Palm Fibers (DPFs): Study of the Interface of DPF–Polyurethane Composite. South Afr. J. Chem. Eng..

[24] Yan L., Chouw N., Yuan X. (2012). Improving the Mechanical Properties of Natural Fibre Fabric Reinforced Epoxy Composites by Alkali Treatment. J. Reinf. Plast. Compos..

[25] Fernandes E.M., Mano J.F., Reis R.L. (2013). Hybrid Cork–Polymer Composites Containing Sisal Fibre: Morphology, Effect of the Fibre Treatment on the Mechanical Properties and Tensile Failure Prediction. Compos. Struct..

[26] Ayeni I.S., Lim N.H.A.S., Samad M. (2024). Engineering Properties of Natural Fibre-Reinforced One-Part Geopolymer Concrete. Constr. Build. Mater..

[27] Abdelmouleh M., Boufi S., Belgacem M.N., Duarte A.P., Ben Salah A., Gandini A. (2004). Modification of Cellulosic Fibres with Functionalised Silanes: Development of Surface Properties. Int. J. Adhes. Adhes..

[28] Gao Y., Chen Y., Gao J., Gao J., Yang L., Zhang J., Nan R., Cai M., Zhang J. (2022). Properties of *Arenga Engleri Becc* Palm Fiber Particles with Silane Coupling Agent KH570 Treatments for Application in Polymer/Cement Composites. J. Nat. Fibers.

[29] Zhou F., Cheng G., Jiang B. (2014). Effect of Silane Treatment on Microstructure of Sisal Fibers. Appl. Surf. Sci..

[30] Asim M., Jawaid M., Abdan K., Ishak M.R. (2016). Effect of Alkali and Silane Treatments on Mechanical and Fibre-Matrix Bond Strength of Kenaf and Pineapple Leaf Fibres. J. Bionic Eng..

[31] Feng Y., Wang Q., Li L., Ma Y., Li X. (2023). Multiscale Analysis of Silane Coupling Agent Modified Rubber-Fiber Concrete Interfaces. Mater. Today Commun..

[32] Wu W., Zuo H. (2016). Used Tire Rubber Powder/Plant Cellulose Composites Treated with Coupling Agent. Cellulose.

[33] Liu T., Wei H., Zhou A., Zou D., Jian H. (2020). Multiscale Investigation on Tensile Properties of Ultra-High Performance Concrete with Silane Coupling Agent Modified Steel Fibers. Cem. Concr. Compos..

[34] Zhao L., Ding Y., Li S., Song Y., Gong H., Zhang Y. (2024). Silane Treatment for Sisal Fibers to Improve the Degradation Resistance and Interface with Cement Matrix. Constr. Build. Mater..

[35] Liu M., Dai W., Li M., Jin W., Yang X., Han Y., Huang M. (2024). Mechanism of Interface Performance Enhancement of Nano-SiO_2_ Modified Polyvinyl Alcohol Fiber Reinforced Geopolymer Concrete: Experiments, Microscopic Characterization, and Molecular Simulation. J. Build. Eng..

[36] Gapsari F., Wijatmiko I., Andoko A., Diharjo K., Mavinkere Rangappa S., Siengchin S. (2024). Modification on Fiber from Alkali Treatment and AESO Coating to Enhance UV-Light and Water Absorption Resistance in Kapok Fiber Reinforced Polyester Composites. J. Nat. Fibers.

[37] Li Y., Ma H., Shen Y., Li Q., Zheng Z. (2015). Effects of Resin inside Fiber Lumen on the Mechanical Properties of Sisal Fiber Reinforced Composites. Compos. Sci. Technol..

[38] Adekunle K.F. (2015). Surface Treatments of Natural Fibres—A Review: Part 1. Open J. Polym. Chem..

[39] Kalia S., Kaith B.S., Kaur I. (2009). Pretreatments of Natural Fibers and Their Application as Reinforcing Material in Polymer Composites—A Review. Polym. Eng. Sci.

[40] Waqas R.M., Butt F., Zhu X., Jiang T., Tufail R.F. (2021). A Comprehensive Study on the Factors Affecting the Workability and Mechanical Properties of Ambient Cured Fly Ash and Slag Based Geopolymer Concrete. Appl. Sci..

[41] Wang H., Wu H., Xing Z., Wang R., Dai S. (2021). The Effect of Various Si/Al, Na/Al Molar Ratios and Free Water on Micromorphology and Macro-Strength of Metakaolin-Based Geopolymer. Materials.

[42] Mohr B.J., Biernacki J.J., Kurtis K.E. (2007). Supplementary Cementitious Materials for Mitigating Degradation of Kraft Pulp Fiber-Cement Composites. Cem. Concr. Res..

[43] Wei J., Meyer C. (2016). Utilization of Rice Husk Ash in Green Natural Fiber-Reinforced Cement Composites: Mitigating Degradation of Sisal Fiber. Cem. Concr. Res..

[44] Gholampour A., Danish A., Ozbakkaloglu T., Yeon J.H., Gencel O. (2022). Mechanical and Durability Properties of Natural Fiber-Reinforced Geopolymers Containing Lead Smelter Slag and Waste Glass Sand. Constr. Build. Mater..

[45] Poletanovic B., Dragas J., Ignjatovic I., Komljenovic M., Merta I. (2020). Physical and Mechanical Properties of Hemp Fibre Reinforced Alkali-Activated Fly Ash and Fly Ash/Slag Mortars. Constr. Build. Mater..

[46] Phoo-ngernkham T., Maegawa A., Mishima N., Hatanaka S., Chindaprasirt P. (2015). Effects of Sodium Hydroxide and Sodium Silicate Solutions on Compressive and Shear Bond Strengths of FA–GBFS Geopolymer. Constr. Build. Mater..

[47] Sachet W.H., Salman W.D. (2021). Geopolymer Concrete, Mortar, and Paste: A Review. IOP Conf. Ser. Mater. Sci. Eng..

[48] Sá Ribeiro M.G., Miranda I.P.A., Kriven W.M., Ozer A., Sá Ribeiro R.A. (2024). High Strength and Low Water Absorption of Bamboo Fiber-Reinforced Geopolymer Composites. Constr. Build. Mater..

[49] Jegan M., Annadurai R., Kannan Rajkumar P.R. (2023). A State of the Art on Effect of Alkali Activator, Precursor, and Fibers on Properties of Geopolymer Composites. Case Stud. Constr. Mater..

[50] Silva G., Castañeda D., Kim S., Castañeda A., Bertolotti B., Ortega-San-Martin L., Nakamatsu J., Aguilar R. (2019). Analysis of the Production Conditions of Geopolymer Matrices from Natural Pozzolana and Fired Clay Brick Wastes. Constr. Build. Mater..

[51] Li H., Tang W., Tan Y., Wang Y., Guo B., Gao P., Zhan B., Yu Q. (2025). Effect of Short Fibers on Non-Uniform Strain Distribution of Geopolymer Mortar under Dry Conditions. Constr. Build. Mater..

[52] Huang Z., Wang Q. (2024). The Effect of Temperature on Dissolution Activity of Fly Ash and Metakaolin in Alkaline Conditions. Cem. Concr. Compos..

[53] Assaedi H., Shaikh F.U.A., Low I.M. (2016). Characterizations of Flax Fabric Reinforced Nanoclay-Geopolymer Composites. Compos. Part B Eng..

[54] Wang X., Zheng Q., Dong S., Ashour A., Han B. (2020). Interfacial Characteristics of Nano-Engineered Concrete Composites. Constr. Build. Mater..

[55] Wang Y.-S., Peng K.-D., Alrefaei Y., Dai J.-G. (2021). The Bond between Geopolymer Repair Mortars and OPC Concrete Substrate: Strength and Microscopic Interactions. Cem. Concr. Compos..

[56] Oriez V., Peydecastaing J., Pontalier P.-Y. (2020). Lignocellulosic Biomass Mild Alkaline Fractionation and Resulting Extract Purification Processes: Conditions, Yields, and Purities. Clean Technol..

[57] Rao J., Zhou Y., Fan M. (2018). Revealing the Interface Structure and Bonding Mechanism of Coupling Agent Treated WPC. Polymers.

[58] Mourak A., Hajjaji M. (2024). Moroccan Heated Clay-Based Geopolymer Reinforced with Date Palm Cellulose: Microstructure Characterization and Mechanical/Physical Properties. Clay Miner..

[59] Sethi S., Ray B.C. (2015). Environmental Effects on Fibre Reinforced Polymeric Composites: Evolving Reasons and Remarks on Interfacial Strength and Stability. Adv. Colloid Interface Sci..

[60] Rahman A.S., Radford D.W. (2017). Evaluation of the Geopolymer/Nanofiber Interfacial Bond Strength and Their Effects on Mode-I Fracture Toughness of Geopolymer Matrix at High Temperature. Compos. Interfaces.

[61] Wang K., Lin B., Wu B., Yao Y. (2024). Repair of Ordinary Concrete Using Basalt Fiber Reinforced Geopolymer: High Temperature Resistance and Micro Structure Evolution of Adhesive Interface. J. Build. Eng..

[62] Yang Y., Xiao Y., Zhou W., Jiskani I.M., Luan B. (2025). Strength Analysis of Geopolymer-Rock Interface: Mohr-Coulomb Shear and Interface Transition Zone (ITZ) Nanoindentation Testing. Constr. Build. Mater..

[63] Silva G., Kim S., Bertolotti B., Nakamatsu J., Aguilar R. (2020). Optimization of a Reinforced Geopolymer Composite Using Natural Fibers and Construction Wastes. Constr. Build. Mater..

[64] Sigrüner M., Muscat D., Strübbe N. (2021). Investigation on pull-out Behavior and Interface Critical Parameters of Polymer Fibers Embedded in Concrete and Their Correlation with Particular Fiber Properties. J. Appl. Polym. Sci..

[65] Ferreira S.R., Pepe M., Martinelli E., De Andrade Silva F., Toledo Filho R.D. (2018). Influence of Natural Fibers Characteristics on the Interface Mechanics with Cement Based Matrices. Compos. Part B Eng..

[66] Liu B., Hu X., Li Y., Xiao T., Xu F. (2020). Internal Three-Dimensional Strain Evolution of the Failure Process for Short Carbon Fiber Composite through in Situ Synchrotron Radiation X-Ray Computed Tomography. Carbon.

[67] Li S., Chen D., Lu Y., Liu Z. (2024). Fatigue Fracture Characteristics of Normal Concrete and High Ductility Geopolymer Bonding Based on DIC Technique. Thin-Walled Struct..

[68] Leite F.L., Herrmann P.S.P., Da Róz A.L., Ferreira F.C., Curvelo A.A.S., Mattoso L.H.C. (2006). Investigation of Sisal Fibers by Atomic Force Microscopy: Morphological and Adhesive Characteristics. J. Nanosci. Nanotech..

[69] Liu W., Shomali A., Zhang C., Coasne B., Carmeliet J., Derome D. (2024). Nanostructure and Interfacial Mechanical Properties of PEG/Cellulose Nanocomposites Studied with Molecular Dynamics. Carbohydr. Polym..

[70] Qin R., Li Y., Wong S.Y., Teo F.Y., Yu Z., Sun L., Zheng Y. (2024). Understanding on the Creep Behavior of Fiber Reinforced Polymer via Fiber/Matrix Interaction. Constr. Build. Mater..

[71] Huang H., Luo J., Peng C., Sun T., Deng T., Hu J., Guzal Anvarovna K., Azizbek Davlatali Ugli N., Hou D., Wei J. (2023). Interfacial Bond between Modified Micro Carbon Fiber and High-Strength Cement Paste in UHPC: Bond-Slip Tests and Molecular Dynamic Simulation. Cem. Concr. Compos..

[72] Alomayri T., Low I.M. (2013). Synthesis and Characterization of Mechanical Properties in Cotton Fiber-Reinforced Geopolymer Composites. J. Asian Ceram. Soc..

[73] Sá Ribeiro R.A., Sá Ribeiro M.G., Sankar K., Kriven W.M. (2016). Geopolymer-Bamboo Composite—A Novel Sustainable Construction Material. Constr. Build. Mater..

[74] Korniejenko K., Frączek E., Pytlak E., Adamski M. (2016). Mechanical Properties of Geopolymer Composites Reinforced with Natural Fibers. Procedia Eng..

[75] Chen R., Ahmari S., Zhang L. (2014). Utilization of Sweet Sorghum Fiber to Reinforce Fly Ash-Based Geopolymer. J. Mater. Sci..

[76] Amalia F., Akifah N., Nurfadilla, Subaer (2017). Development of Coconut Trunk Fiber Geopolymer Hybrid Composite for Structural Engineering Materials. IOP Conf. Ser. Mater. Sci. Eng..

[77] Zhao C., Wang Z., Zhu Z., Guo Q., Wu X., Zhao R. (2023). Research on Different Types of Fiber Reinforced Concrete in Recent Years: An Overview. Constr. Build. Mater..

[78] Zulfiati R., Saloma, Idris Y. (2019). Mechanical Properties of Fly Ash-Based Geopolymer with Natural Fiber. J. Phys. Conf. Ser..

[79] Ayawanna J., Poowancum A. (2024). Enhancing Flexural Strength of Metakaolin-Based Geopolymer Reinforced with Different Types of Fibers. Sustain. Chem. Pharm..

[80] Ranjbar N., Zhang M. (2020). Fiber-Reinforced Geopolymer Composites: A Review. Cem. Concr. Compos..

[81] Alzeer M., MacKenzie K. (2013). Synthesis and Mechanical Properties of Novel Composites of Inorganic Polymers (Geopolymers) with Unidirectional Natural Flax Fibres (*Phormium Tenax*). Appl. Clay Sci..

[82] Addis L.B., Sendekie Z.B., Habtu N.G., Schubert D.W., Roether J.A., Boccaccini A.R. (2024). False Banana Fiber Reinforced Geopolymer Composite—A Novel Sustainable Material. Ceram. Int..

[83] Alomayri T., Shaikh F.U.A., Low I.M. (2013). Thermal and Mechanical Properties of Cotton Fabric-Reinforced Geopolymer Composites. J. Mater. Sci..

[84] Alomayri T., Shaikh F.U.A., Low I.M. (2013). Characterisation of Cotton Fibre-Reinforced Geopolymer Composites. Compos. Part B Eng..

[85] Wongsa A., Kunthawatwong R., Naenudon S., Sata V., Chindaprasirt P. (2020). Natural Fiber Reinforced High Calcium Fly Ash Geopolymer Mortar. Constr. Build. Mater..

[86] Alomayri T., Assaedi H., Shaikh F.U.A., Low I.M. (2014). Effect of Water Absorption on the Mechanical Properties of Cotton Fabric-Reinforced Geopolymer Composites. J. Asian Ceram. Soc..

[87] Farhan K.Z., Johari M.A.M., Demirboğa R. (2021). Impact of Fiber Reinforcements on Properties of Geopolymer Composites: A Review. J. Build. Eng..

[88] Poletanovic B., Janotka I., Janek M., Bacuvcik M., Merta I. (2021). Influence of the NaOH-Treated Hemp Fibres on the Properties of Fly-Ash Based Alkali-Activated Mortars Prior and after Wet/Dry Cycles. Constr. Build. Mater..

[89] Chen C., Zhang X., Hao H. (2023). Dynamic Tensile Properties of Geopolymer Concrete and Fibre Reinforced Geopolymer Concrete. Constr. Build. Mater..

[90] Yang S., Zhao R., Ma B., Si R., Zeng X. (2023). Mechanical and Fracture Properties of Fly Ash-Based Geopolymer Concrete with Different Fibers. J. Build. Eng..

[91] Low I.M., McGrath M., Lawrence D., Schmidt P., Lane J., Latella B.A., Sim K.S. (2007). Mechanical and Fracture Properties of Cellulose-Fibre-Reinforced Epoxy Laminates. Compos. Part A Appl. Sci. Manuf..

[92] Wambua P., Ivens J., Verpoest I. (2003). Natural Fibres: Can They Replace Glass in Fibre Reinforced Plastics?. Compos. Sci. Technol..

[93] Rayapudi S., Rao T.C.S., Pancharathi R.K., Leung K.Y., Chandra C., Kishen J.M. (2024). Strength Characteristics and Impact Resistance of Fiber-Reinforced Geopolymer Concrete Elements. Low Carbon Materials and Technologies for a Sustainable and Resilient Infrastructure.

[94] Md B., Unnikrishnan S. (2022). Mechanical Strength and Impact Resistance of Hybrid Fiber Reinforced Concrete with Coconut and Polypropylene Fibers. Mater. Today Proc..

[95] Wang W., Zhang Y., Mo Z., Chouw N., Jayaraman K., Xu Z. (2023). A Critical Review on the Properties of Natural Fibre Reinforced Concrete Composites Subjected to Impact Loading. J. Build. Eng..

[96] Santana H.A., Andrade Neto J.S., Ribeiro D.V., Cilla M.S., Dias C.M.R. (2021). Accelerated Alkaline Attack of 3D Printing Polymers to Assess Their Durability in Geopolymer-Based Matrices. J. Mater. Civ. Eng..

[97] Li F., Chen D., Lu Y., Zhang H., Li S. (2022). Influence of Mixed Fibers on Fly Ash Based Geopolymer Resistance against Freeze-Thaw Cycles. J. Non-Cryst. Solids.

[98] Silva G., Kim S., Aguilar R., Nakamatsu J. (2020). Natural Fibers as Reinforcement Additives for Geopolymers—A Review of Potential Eco-Friendly Applications to the Construction Industry. Sustain. Mater. Technol..

[99] Zhao N., Wang S., Quan X., Xu F., Liu K., Liu Y. (2021). Behavior of Polyvinyl Alcohol Fiber Reinforced Geopolymer Composites under the Coupled Attack of Sulfate and Freeze-Thaw in a Marine Environment. Ocean Eng..

[100] Bariş K.E., Tanaçan L. (2023). Durability behavior of banana fiber-reinforced natural pozzolan geopolymer. J. Green Build..

[101] Gencel O., Yavuz Bayraktar O., Kaplan G., Benli A., Martínez-Barrera G., Brostow W., Tek M., Bodur B. (2021). Characteristics of Hemp Fibre Reinforced Foam Concretes with Fly Ash and Taguchi Optimization. Constr. Build. Mater..

[102] Tan J., Huang Y., Liu L., Yu S., Zheng G., Dobrotă D., Cheng C. (2021). Preparation and Freeze-Thaw Resistance of Geopolymer-Based Natural Plant Fiber Composites. Advances in Transdisciplinary Engineering.

[103] Xuan X., Tan J., Huang Y., Xie M., Zheng G. (2020). Preparation and Freeze Resistance of Geopolymer-Bagasse Fiber Composites. Bull. Chin. Ceram. Soc.

[104] Sahraei Moghadam A., Mirza Goltabar Roshan A., Omidinasab F. (2023). Utilization of Agricultural Wastes as Fiber, Binder and Aggregates of Geopolymer Mortars: Application of Taguchi Method for Strength and Durability Optimization. J. Build. Eng..

[105] Nkwaju R.Y., Djobo J.N.Y., Nouping J.N.F., Huisken P.W.M., Deutou J.G.N., Courard L. (2019). Iron-Rich Laterite-Bagasse Fibers Based Geopolymer Composite: Mechanical, Durability and Insulating Properties. Appl. Clay Sci..

[106] Yanou R.N., Kaze R.C., Adesina A., Nemaleu J.G.D., Jiofack S.B.K., Djobo J.N.Y. (2021). Performance of Laterite-Based Geopolymers Reinforced with Sugarcane Bagasse Fibers. Case Stud. Constr. Mater..

[107] Batista Dos Santos G.Z., Passos De Oliveira D., De Almeida Melo Filho J., Marques Da Silva N. (2021). Sustainable Geopolymer Composite Reinforced with Sisal Fiber: Durability to Wetting and Drying Cycles. J. Build. Eng..

[108] Noor Abbas A.-G., Nora Aznieta Abdul Aziz F., Abdan K., Azline Mohd Nasir N., Fahim Huseien G. (2023). Experimental Study on Durability Properties of Kenaf Fibre-Reinforced Geopolymer Concrete. Constr. Build. Mater..

[109] Wei Y., Chai J., Qin Y., Li Y., Xu Z., Li Y., Ma Y. (2021). Effect of Fly Ash on Mechanical Properties and Microstructure of Cellulose Fiber-Reinforced Concrete under Sulfate Dry–Wet Cycle Attack. Constr. Build. Mater..

[110] Khan M., Cao M., Chu S.H., Ali M. (2022). Properties of Hybrid Steel-Basalt Fiber Reinforced Concrete Exposed to Different Surrounding Conditions. Constr. Build. Mater..

[111] Gulzar M.A., Ali B., Barakat O., Azab M., Najemalden A.M., Salih Mohammed A., Alashker Y. (2023). Influence of Jute Fiber on Tensile, Electrical, and Permeability Characteristics of Slag Concrete: A Better, Cheaper, and Eco-Friendly Substitute for Conventional Concrete. J. Nat. Fibers.

[112] Hadigheh S.A., Ke F., Fatemi H. (2022). Durability Design Criteria for the Hybrid Carbon Fibre Reinforced Polymer (CFRP)-Reinforced Geopolymer Concrete Bridges. Structures.

[113] Zhang X., Long K., Liu W., Li L., Long W.-J. (2020). Carbonation and Chloride Ions’ Penetration of Alkali-Activated Materials: A Review. Molecules.

[114] Ye H., Radlińska A. (2017). Carbonation-Induced Volume Change in Alkali-Activated Slag. Constr. Build. Mater..

[115] Pasupathy K., Sanjayan J., Rajeev P. (2021). Evaluation of Alkalinity Changes and Carbonation of Geopolymer Concrete Exposed to Wetting and Drying. J. Build. Eng..

[116] Huang Y., Tan J., Xuan X., Wei S., Liu L., Yu S., Zheng G. (2022). Durability of Plant Fiber Reinforced Alkali Activated Composites. Constr. Build. Mater..

[117] Merta I., Poletanovic B., Dragas J., Carevic V., Ignjatovic I., Komljenovic M. (2022). The Influence of Accelerated Carbonation on Physical and Mechanical Properties of Hemp-Fibre-Reinforced Alkali-Activated Fly Ash and Fly Ash/Slag Mortars. Polymers.

[118] Pouhet R., Cyr M. (2015). Alkali–Silica Reaction in Metakaolin-Based Geopolymer Mortar. Mater. Struct..

[119] Ardanuy M., Claramunt J., Toledo Filho R.D. (2015). Cellulosic Fiber Reinforced Cement-Based Composites: A Review of Recent Research. Constr. Build. Mater..

[120] Zhang B. (2024). Durability of Low-Carbon Geopolymer Concrete: A Critical Review. Sustain. Mater. Technol..

[121] Zhou B., Wang L., Ma G., Zhao X., Zhao X. (2020). Preparation and Properties of Bio-Geopolymer Composites with Waste Cotton Stalk Materials. J. Clean. Prod..

[122] Lezzerini M., Aquino A., Pagnotta S. (2024). Acid Resistance of Metakaolin-Based Geopolymers and Geopolymeric Mortars Reinforced with Coconut Fibers. Fibers.

[123] Thanushan K., Sathiparan N. (2022). Mechanical Performance and Durability of Banana Fibre and Coconut Coir Reinforced Cement Stabilized Soil Blocks. Materialia.

[124] Sá Ribeiro M.G., Sá Ribeiro M.G., Keane P.F., Sardela M.R., Kriven W.M., Sá Ribeiro R.A. (2021). Acid Resistance of Metakaolin-Based, Bamboo Fiber Geopolymer Composites. Constr. Build. Mater..

[125] Li Z., Zhang S., Zuo Y., Chen W., Ye G. (2019). Chemical Deformation of Metakaolin Based Geopolymer. Cem. Concr. Res..

[126] Alcivar-Bastidas S., Petroche D.M., Cornejo M.H., Martinez-Echevarria M.J. (2024). Effect of Aging Process on Mechanical Performance of Reinforced Mortar with NaOH Abaca Fibers. Case Stud. Constr. Mater..

[127] Haily E., Zari N., Bouhfid R., Qaiss A. (2023). Natural Fibers as an Alternative to Synthetic Fibers in the Reinforcement of Phosphate Sludge-Based Geopolymer Mortar. J. Build. Eng..

[128] Ranjithkumar M.G., Chandrasekaran P., Rajeshkumar G. (2022). Characterization of Sustainable Natural Fiber Reinforced Geopolymer Composites. Polym. Compos..

[129] Kawashima S., Shah S.P. (2011). Early-Age Autogenous and Drying Shrinkage Behavior of Cellulose Fiber-Reinforced Cementitious Materials. Cem. Concr. Compos..

[130] Lv C., Liu J. (2023). Alkaline Degradation of Plant Fiber Reinforcements in Geopolymer: A Review. Molecules.

[131] Xu S., Wu C., Yue J., Xu Z. (2022). Shrinkage and Mechanical Properties of Fibre-Reinforced Blast Furnace Slag-Steel Slag-Based Geopolymer. Adv. Civ. Eng..

[132] Amran M., Onaizi A.M., Makul N., Abdelgader H.S., Tang W.C., Alsulami B.T., Alluqmani A.E., Gamil Y. (2023). Shrinkage Mitigation in Alkali-Activated Composites: A Comprehensive Insight into the Potential Applications for Sustainable Construction. Results Eng..

[133] Walbrück K., Drewler L., Witzleben S., Stephan D. (2021). Factors Influencing Thermal Conductivity and Compressive Strength of Natural Fiber-Reinforced Geopolymer Foams. Open Ceram..

[134] Novais R.M., Carvalheiras J., Senff L., Lacasta A.M., Cantalapiedra I.R., Giro-Paloma J., Seabra M.P., Labrincha J.A. (2020). Multifunctional Cork—Alkali-Activated Fly Ash Composites: A Sustainable Material to Enhance Buildings’ Energy and Acoustic Performance. Energy Build..

[135] Silva G., Salirrosas J., Ruiz G., Kim S., Nakamatsu J., Aguilar R. (2019). Evaluation of Fire, High-Temperature and Water Erosion Resistance of Fiber-Reinforced Lightweight Pozzolana-Based Geopolymer Mortars. IOP Conf. Ser. Mater. Sci. Eng..

[136] Ma G., Li Z., Wang L., Wang F., Sanjayan J. (2019). Mechanical Anisotropy of Aligned Fiber Reinforced Composite for Extrusion-Based 3D Printing. Constr. Build. Mater..

[137] Liu J., Lv C. (2022). Properties of 3D-Printed Polymer Fiber-Reinforced Mortars: A Review. Polymers.

[138] Kozub B., Gądek S., Tyliszczak B., Wojnar L., Korniejenko K. (2024). Leveraging 3D Printing Capability for Geopolymer Composites Based on Fly Ash with Cotton Fibers Addition. Int. J. Eng. Technol. Innov..

[139] Kong X., Dai L., Wang Y., Qiao D., Hou S., Wang S. (2022). Influence of Kenaf Stalk on Printability and Performance of 3D Printed Industrial Tailings Based Geopolymer. Constr. Build. Mater..

